# The Effects of Environmental Factors on General Human Health: A Scoping Review

**DOI:** 10.3390/healthcare12212123

**Published:** 2024-10-24

**Authors:** Amina Sundas, Ivan Contreras, Omer Mujahid, Aleix Beneyto, Josep Vehi

**Affiliations:** 1 Modeling & Intelligent Control Engineering Laboratory, Institut d’Informatica i Applicacions, Universitat de Girona, 17003 Girona, Spain; amina.sundas@udg.edu (A.S.); omer.mujahid@udg.edu (O.M.); aleix.beneyto@udg.edu (A.B.); josep.vehi@udg.edu (J.V.); 2 Centro de Investigación Biomédica en Red de Diabetes y Enfermedades Metabólicas Asociadas (CIBERDEM), 17003 Girona, Spain

**Keywords:** environmental factors, human health, pollution, geographical location, Copernicus, meteorological factors

## Abstract

**Background/Objectives:** The external environment constantly influences human health through many factors, including air quality, access to green spaces, exposure to pollutants, and climate change. Contamination poses a substantial threat to human well-being; conversely, environmental factors also positively impact health. The purpose of this study is to provide a comprehensive review of the complex relationship between various environmental factors and human health. While individual studies have explored specific aspects, a broader integrative understanding is lacking. **Methods:** Through databases (PubMed, Cochrane, Copernicus), 4888 papers were identified, with 166 selected for detailed analysis. **Results:** We summarized recent research, identifying multiple associations between environmental factors such as air pollution, climate change, solar radiation, and meteorological conditions and their impact on various health outcomes, including respiratory, cardiovascular, metabolic and gastrointestinal, renal and urogenital, neurological and psychological health, infectious and skin diseases, and major cancers. We use chord diagrams to illustrate these links. We also show the interaction between different environmental factors. Findings begin with exploring the direct impact of environmental factors on human health; then, the interplay and combined effects of environmental factors, elucidating their (often indirect) interaction and collective contribution to human health; and finally, the implications of climate change on human health. **Conclusions:** Researchers and policymakers need to consider that individuals are exposed to multiple pollutants simultaneously, the “multipollutant exposure phenomenon”. It is important to study and regulate environmental factors by considering the combined impact of various pollutants rather than looking at each pollutant separately. We emphasize actionable recommendations and solutions.

## 1. Introduction

Human health and well-being are strongly correlated with each other. Neglecting environmental elements will have an impact on illness prevention and control, including viral pandemics [1]. According to the WHO, clean air, a stable climate, adequate water, sanitation and hygiene, safe use of chemicals, protection from radiation, healthy and safe workplaces, sound agricultural practices, health-supportive cities and built environments, and a preserved nature are all prerequisites for good health. Humans live in habitats that are intricately exposed to various combinations of natural and synthetic chemicals, social health factors, lifestyle choices, and weather conditions, as well as in various geographic regions to which they have evolved to varying degrees. The ways a human body is exposed to various environmental agents are numerous and it is next to impossible to group all these factors together based on some common ground. Riggs et al. [2] found that the different types of environmental exposures are more variable than an individual’s genetics. Unlike genomes, which are composed of the same chemical entity (DNA), environmental exposures belong to distinct categories that cannot be readily grouped. This makes defining the human environment an extremely challenging task. This may be especially relevant when evaluating how the environment’s numerous interconnected and interdependent components may affect people’s health individually and jointly.

In order to provide a conceptual framework for comprehending how environmental elements affect human health and to assess the contribution of various environmental aspects, a comprehensive description of the environment and its components is required. There have been various reviews and research studies that have investigated at least one of the following relationships: a specific environmental factor and its effects on human health, a combination of environmental factors and their effect on a specific pathology, the relationship between environmental factors and human health in a specific setting or geography, and the inter-effect of two or more environmental factors causing different diseases in humans [3,4]. However, to the best of our knowledge, the effect of the environment as a combination of various environmental factors on issues related to the broad spectrum of human health has not been explored yet. One reason for this is the ambiguity in the methodological rigor with which such a review should be conducted [5]. Moreover, the scope of a review like this is extremely vast and it is a challenging task to conduct a review on such a broad scale.

In the contemporary world, where the natural environment faces many challenges in order to sustain itself, a bigger picture of the environmental issues in light of human health must be discussed. With various forms of pollutants contaminating the existing resources on the planet, humankind’s biggest challenge is identifying the links between these pollutants and general human health. This review aspires to be one such guide to readers by providing a broader view of the environmental issues and the health problems these pollutants cause in human beings. It also dwells on the various resources that could be used to develop an understanding of the relationship between human health and the surrounding environment. New tools and research are urgently required to advance environmental health literacy [6].

Current approaches for studying environmental effects on human health are mostly based on social determinants of health, such as the psychosocial, socioeconomic, demographic, psychological, geographical, and the body’s internal chemical environment. However, these models are limited by their exclusive focus on a specific domain of the environment or a particular health issue. In addition, social determinants of health do not consider the natural (ecological and geographic) aspects of the human environment or personal microenvironments, which frequently vary among people living under the same societal conditions [7]. Additionally, these models mainly rely on studying each exposure separately, which prevents a collective assessment of several interacting environmental domains. Although frameworks like the socio-exposome framework integrate social and environmental factors to understand their combined impact on health, these frameworks have yet to be widely adopted because of the involvement of a complex interplay of multiple exposures, requiring data from diverse fields such as epidemiology, sociology, environmental science, and public health. This interdisciplinary approach can be challenging to manage and integrate effectively, leading to difficulties in operationalizing the framework [8]. As defined by Riggs et al. [2], environmental exposures, collectively referred to as “exposome”, fall into three categories: internal, specific external, and general external. The internal environment comprises the body’s internal chemical environment, including chemicals produced by metabolism, physical activity, gut microflora, inflammation, and oxidative stress. Specific external exposures encompass environmental pollutants, infectious diseases, diet, alcohol, and tobacco smoke. General external exposures include socioeconomic status, psychological influences, the built environment, and climate. The published articles in the domain of environmental health discuss one or more variants of these environmental exposures.

The interplay between these domains highlights the complex and interconnected nature of the exposome. For instance, internal exposures such as inflammation and oxidative stress can be influenced by specific external factors like diet and pollution, as well as general external factors like socioeconomic status and psychological stress. Pollutants and dietary habits can exacerbate oxidative stress and inflammation, which are internal responses to these external exposures. Moreover, socioeconomic status and the built environment can affect diet quality and exposure to pollutants, further influencing internal chemical processes and overall health outcomes. Psychological influences, shaped by social and economic conditions, can impact physical activity levels and metabolic processes, creating a dynamic interaction between internal, specific external, and general external factors.

The purpose of this study is to provide a comprehensive review of the various environmental factors impacting human health. While many studies have investigated individual factors or specific health outcomes, this review aims to synthesize the existing literature to better understand the collective impact of multiple environmental factors on human health. For this goal, the review needs to identify associations/links between the external environment and human health issues, and the focus is solely on the environmental factors that have a major effect on human health. The term “major” is used to highlight the level of importance and influence these factors have due to their wide-reaching consequences. This work is carried out to establish a general understanding of the adverse effects of the external environment on human health. It is understood that almost every human being suffers from some kind of health disorder or disease, and for each of these disorders or diseases, there is a great number of medical terminologies based on different contexts [9]. 

Furthermore, when medical terms link disease causes to environmental factors, their complexity and scope increase significantly. This means that these terminologies not only describe diseases but also account for how environmental influences contribute to their development. To comprehend environmental health issues broadly, it is essential to categorize them systematically. This grouping enables us to gain a comprehensive overview of how health and environmental factors are interconnected. The selection of major human health issues in this review was based on the importance of the respective health issue in terms of categorization applied by other authors [10,11], the amount of evidence found in the literature for a specific health issue in the context of environmental effects, and the overall global impact of the disease [12,13]. Moreover, a person’s health depends upon several factors. That is why, from an individual’s perspective, the term “health” needs to be categorized in such a way that it would cover major factors that are extremely important for one to be considered a healthy human being.

## 2. Materials and Methods

**Figure 1:**
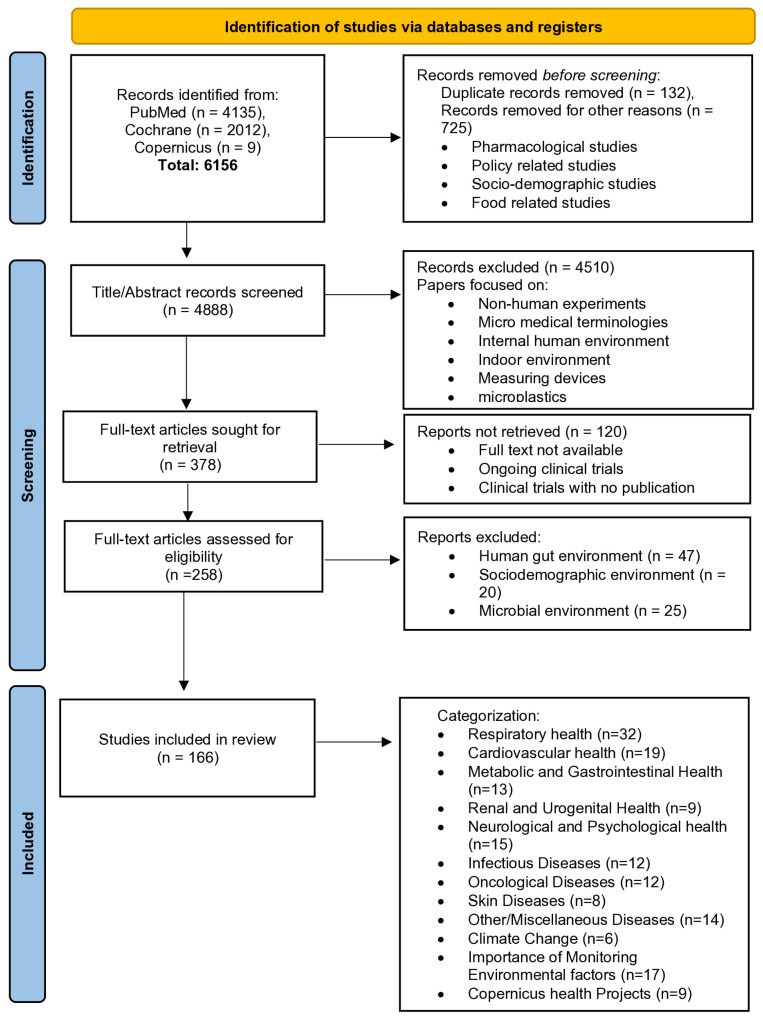
Overview of the review process and classification of the literature using PRISMA 2020 flow diagram guidelines.

The manuscripts were searched using the advanced search functionality on PubMed and Cochrane, and the search functionality on the Copernicus website. A pool of 5702 manuscripts was obtained from PubMed and Cochrane by combining the results of multiple individual keyword searches. PubMed was chosen for the manuscript search because it is considered one of the best sources within the scientific literature for biomedical and health sciences [14], whereas Cochrane was chosen to obtain manuscripts based on clinical trials to increase the diversity of our article pool and expand the horizon of our literature review. In this review, articles published in English over the past 10 years (from the year 2023) were taken into consideration. Moreover, Copernicus was chosen to obtain the literature about the projects based on Copernicus’ earth observatory data. We selected nine Copernicus health-related projects for review. This review adheres to the PRISMA (Preferred Reporting Items for Systematic Reviews and Meta-Analyses) guidelines for scoping reviews. The study selection process is detailed in Figure 1, including the initial manuscript search, screening, and the final inclusion of articles based on pre-defined eligibility criteria.

The following keywords were searched in PubMed and Cochrane; the keywords used along with the selected papers against the total obtained results (selected papers/total obtained results) were as follows: cancer AND ecological study (19/411), allergies AND air pollution AND environmental factors (36/1206), health AND environmental factors (62/1459), health AND geographical information (7/23), respiratory health AND environmental factors (28/175), cardiovascular health AND environmental factors (29/1348), digestive AND environmental factors (8/174), mental health AND environmental factors (26/906), gastrointestinal OR metabolic health AND (environmental factors) (1/36), renal OR urogenital health AND environmental factors (10/71), skin AND environmental factors (5/129), rheumatology AND environmental factors (6/79), otolaryngology AND environmental factors (4/63), ophthalmology AND environmental factors (4/67).

The initial search identified 6156 records from different individual keyword searches (Figure 1). The titles and abstracts of 4888 manuscripts were assessed and 4510 articles were removed following title and abstract screening. We found that most of the obtained articles were related to the internal human environment, indoor environment, non-human experiments, micro-medical terminologies, or measuring devices. This screening resulted in 378 papers that were screened for full text. Here, articles that were not possible to retrieve or include in the review were removed. These articles included those that did not have full text available, were ongoing clinical trials, or trials with no publication reporting the findings. A total of 258 articles were shortlisted for full-text eligibility after this phase. A thorough inspection of these articles resulted in the exclusion of 92 articles that were based on human genomes and gut bacteria environment, socio-demographic environment, and microbial environment. Finally, a total of 166 articles underwent a full-text review. This collection was then broken down into several categories, which were designed to facilitate the grouping of studies according to both shared and specific characteristics. The classification includes nine human health categories, Copernicus health projects (provided in Supplementary File S1), articles that were focused on the effect of climate change on human health, and articles with examples showing the importance of monitoring environmental factors. 

## 3. Results

**Figure 2:**
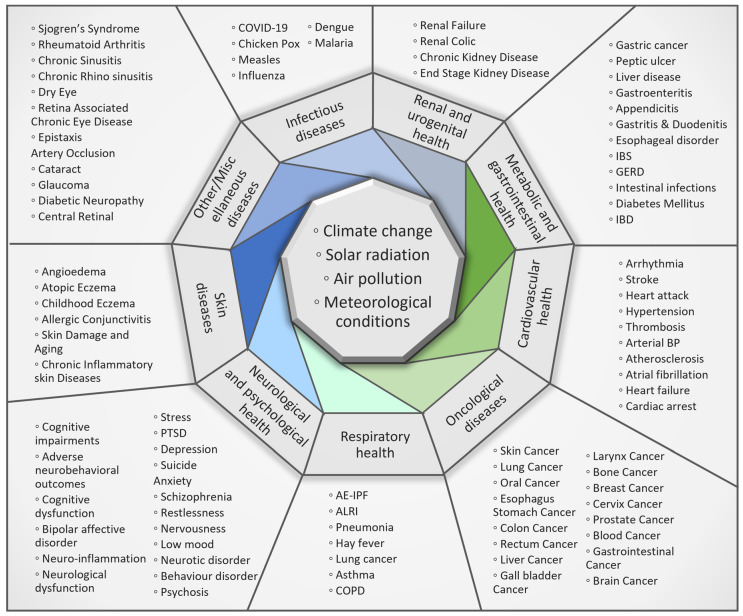
Impacts of major environmental factors (external environment) on human health (nine major categories with several diseases) (Abbreviations: COPD: chronic obstructive pulmonary disorder, IBS: irritable bowel syndrome, IBD: inflammatory bowel disease, GERD: PTSD: post-traumatic stress disorder, AE-IPF: acute exacerbation of idiopathic pulmonary fibrosis, ALRI: acute lower respiratory infections, BP: blood pressure).

Human health was divided into nine main categories for the purpose of this review: cardiovascular health, respiratory health, metabolic and gastrointestinal health, renal or urogenital health, neurological and psychological health, infectious diseases, skin diseases, oncological diseases, and other/miscellaneous diseases. These categories are analyzed in light of the various diseases they comprise and are discussed in the following nine subsections. Each of the subsections presents the literary evidence found that shows the relationship between environmental factors (mainly air pollution, greenness, and weather) and the proposed categories of human health issues. Figure 2 shows a diagram of the impacts of major environmental factors on human health discussed in this review. From this, we developed a conjecture about the state of a healthy human being, whereby a person is considered healthy if their cardiovascular, respiratory, and digestive systems are in good condition along with the person possessing mental well-being and being protected from various diseases. A similar approach is adopted for the categorization of major environmental factors. A detailed summary and categorization of the manuscripts/projects used in this review is included in the Supplementary Information (Table S1).

Our environment consists of several factors such as air, water, soil, climate, space, urban built, and weather. All of these contribute simultaneously to our health. Since the human body’s exposure to the external environment happens in a variety of ways, and the environment itself consists of numerous variables, writing down a combination of all the ways that human environmental exposure can occur results in a long list of categories. We have categorized the environmental variables into groups that contain only the major environmental factors that affect human health. The environmental factors in this review are limited to air pollution, climate change, solar radiation, and meteorological conditions. The results are presented as subsections presenting literary evidence of environmental health for each of the five shortlisted health categories. Subsequently, climate change and environmental interplay affecting human health are discussed.

This work highlights both short-term and long-term health effects of environmental exposures. Short-term impacts include immediate or acute health effects, such as respiratory problems due to air pollution, while long-term impacts refer to chronic conditions or diseases, such as cancer or cardiovascular diseases, that may arise from prolonged exposure to environmental hazards. Health problems resulting from environmental factors do not only impact individual well-being but also have profound consequences for society as a whole. Poor health among populations can lead to a decrease in productivity, increased healthcare costs, and a greater burden on healthcare systems [7,15,16,17,18,19]. 

### 3.1. Respiratory Health

One of the main effects of the external environment on general human health is its effect on the respiratory system. From serious asthma exacerbations to those as fatal as lung cancer, different environmental factors have different levels of association with these diseases depending upon geographical locations and seasons. Environmental air pollution is a major health hazard and is responsible for human mortality and morbidity, increased emergency visits, hospitalizations due to acute effects on cardiopulmonary health, and worsening lung function. According to The Lancet Commission on Pollution and Health, air pollution was responsible for at least 16% of global deaths in 2015 and was the leading cause of preventable mortality worldwide [20]. Moreover, the more recent Lancet report of 2019 shows that air pollution is still responsible for the greatest number of deaths among pollution-related mortalities, with 11.65% of the total [12]. 

Air pollution contains greenhouse gases (carbon dioxide (CO_2_), methane (CH_4_), and nitrous oxide (N_2_O)), which contribute to global warming, causing an adverse joint risk factor on human health. Air pollution also includes elements such as nitrogen dioxide (NO_2_), sulfur dioxide (SO_2_), ozone (O_3_), particulate matter (PM) up to 10 µg/m^3^ in size (PM_2.5_–PM_10_), carbon monoxide (CO), and many others. Santos et al. [21] found that air pollution along with household pollution is ranked as the fifth leading risk factor of death worldwide. Air pollution is characterized as a carcinogen by the International Agency for Research on Cancer [22], and inhaling it has a direct effect on human health, either in the short term, where it can aggravate existing respiratory or even cardiovascular complications or in the long-term, where it is linked to lung cancer and susceptibility to respiratory tract infections. It causes oxidative stress and inflammation, which promote tumors. Thomas et al. found that exposure to air pollution is associated with lung cancer regardless of smoking habits, which are considered the main cause of lung cancer [23]. So et al. reported that long-term air pollution exposure is correlated with mortality from cardiovascular diseases, respiratory diseases, and lung cancer [24].

The long-term effects of air pollution are perceived as greater than the short-term effects. However, it is important to note that the impact of air pollution can vary significantly based on the source and exposure duration. In a study by Aix et al., it was found that reductions in PM_2.5_ levels would result in a 2% decrease in the risk of lung cancer, a 3.3% decrease in the risk of mortality, and an 8% decrease in the risk of low birth weight. It is widely accepted in the scientific community that there is no safe level of PM_2.5_ exposure. Research has shown that even low levels of PM_2.5_ can have adverse health effects. Similarly, lower NO_2_ emissions produced a decrease of 0.96% in the risk of mortality [25]. A study by Lefler et al. assessing the association between all-cause mortality and PM_2.5_ and PM_10_ found that among all pollutants, PM_2.5_ mortality associations were the strongest [26]. They also found that exposure to SO_2_ increased the risk of all-cause mortality. The nasopharyngeal (NP) microbiota, which plays an important role in respiratory health, and PM_2.5_ were found to be among the most important environmental factors affecting the composition and community structure of NP flora [27].

The coronavirus pandemic caused by SARS-CoV-2 provided additional evidence of the impact of air pollution on human health [28]. During the lockdown, PM_2.5_ concentrations decreased on average by 29.7% in China and by 17.1% in Europe compared with pre-pandemic daily concentrations in 2016–2019. This decrease prevented an estimated 24,200 premature deaths in China between 1 February and 31 March 2020 and 2190 premature deaths in Europe between 21 February and 17 May 2020 [28]. This analysis showed that substantial health benefits could be achieved once stringent emission control regulations are implemented. 

Air pollution with high levels of PM_2.5_ and PM_10_ is considered to have a close association with asthma exacerbations, which are episodes of worsening asthma symptoms and lung function. Mo Yanga et al. showed that long-term PM_2.5_ and PM_1_ exposures contributed to lung function impairment in children [29]. While exploring the effect of weather factors on an increased number of hospitalizations due to asthma exacerbations in Qingdao (China), Wei et al. found that air pollutants increase the number of emergency visits due to asthma exacerbations [30]. Similarly, in a study by Khatri et al. in Cleveland, Ohio, higher PM levels (PM_2.5_, PM_10_) and NO_2_ levels were evaluated over the short-term and long-term and were found to be positively associated with increased frequency of inflammation from asthma and emergency department visits by children [31]. 

Wang et al. from Taiwan found that children exposed to non-methane hydrocarbons (NMHCs) and methane (CH_4_) hydrocarbons in air pollution had an increased risk of atopic dermatitis. Atopic dermatitis in childhood can lead to hay fever or asthma later. Approximately 15–30% of children and 10% of adults worldwide suffer from it [32]. Higher levels of PM_2.5_ are found in major developing countries like Bangladesh, China, and India. Xing et al. have reported a higher risk of asthma, hay fever, eczema, and food allergies among the population in these countries [33]. Dan Norbäck et al. found that temperature, NO_2_, and PM_10_ are associated with eczema and can also increase the risk of asthma, wheezing, and allergenic rhinitis in children [34]. Tahara et al. suggested that when PM_2.5_ concentrations are increased, exposed idiopathic pulmonary fibrosis (IPF) patients had only a 1 to 2-month time window before diagnosis of acute exacerbations. IPF is a condition in which the lungs become scarred and breathing becomes increasingly difficult [35].

Pablo et al. analyzed associations between outdoor air pollution and the prevalence of asthma in children from Latin America and the Caribbean and found significant associations [36]. Similarly, Yang et al. [37] analyzed, in the Hangzhou region, the relationship between childhood asthma hospital visits and PM, and positive associations were found between both PM_2.5_ and PM_10_ concentrations and emergency department visits for childhood asthma. Shin Yamazaki et al. reported that increased primary care visits at night due to asthma attacks were associated with high ozone levels in the spring and winter seasons [38]. Jo et al. described the association of high concentrations of PM_10_ and O_3_ with increased use of medical care for asthma and chronic obstructive pulmonary disease (COPD) [39]. Similarly, Shin et al. found positive associations between COPD and air pollution exposure. They also specified that PM_2.5_ is a modifiable risk factor for COPD [15]. 

Dąbrowiecki et al. demonstrated how air pollution interacting with the weather had a short-term effect on all-cause mortality and pneumonia-related hospitalizations [28]. A Spanish research study by Alvaro et al. found that lower temperature, higher relative humidity, higher NO_2_ levels, higher SO_2_ levels, higher O_3_ levels, and higher PM_2.5_ levels were associated with increased hospital admissions due to acute lower respiratory infections (ALRIs), which includes pneumonia, bronchitis, and acute bronchitis [40]. In addition, in a sub-Saharan Africa study among children, Graciela et al. showed a significant decline in lung function at modestly high NO and NO_2_ levels and suggested further control of exposure [41]. Allergic rhinitis, also called hay fever, is a major global illness and has been increasing in prevalence in recent years, affecting 10–40% of the global population. Wu et al. found that PM_2.5_, PM_10_, NO_2_, and SO_2_ were all positively associated with hay fever [42].

The ability of urban trees to mitigate air pollution has led some to hypothesize that they could benefit respiratory health; however, the link between tree pollen and the exacerbation of asthma has raised concerns that urban trees could instead exert a negative effect [43]. Greenness encourages active lifestyles, and its health benefits are numerous. It is associated with better mental health, cardiovascular health, and especially immunity. Exposure to microbes, vegetation, soil, and pollen promotes immune health and decreases overall mortality. This exposure also reduces air pollution and can protect one from extreme temperatures. Yang et al. reported various health benefits of greenness [43].

However, in studies like [44], Zhao et al. have also found that food allergies like peanut and egg allergies were increased with increased exposure to greenness; this is due to a type of cross-reactivity between pollen and food sensitization. Furthermore, studies show positive associations between too much exposure to greenness and asthma and hay fever, which is very common worldwide. Stas et al. presented that over the last few years, pollen-related allergies have been increasing through the combined effects of urbanization, climate change, and air pollution. Urbanization and air pollution both alter the chemical composition of pollen and intensify its allergenic potential [45]. Similarly, a large cohort study in Canada emphasized the negative association between air pollution and greenness. The authors Crouse et al. concluded that humans who live closer to greenness are less prone to mortality from air pollution, as greenness attenuates the effects of air pollution, specifically PM_2.5_ [16].

Air temperature affects the human body greatly: both low and high ambient air temperatures are associated with mortality and morbidity. Exposure to adverse ambient air temperature is associated with high morbidity of respiratory diseases. Ma et al. concluded [10] that diurnal temperature ranges (DTRs), which represent the difference between the highest and lowest air temperatures during a single day, were positively correlated with both respiratory and cardiovascular mortality and morbidity. Further, when temperatures either rose or fell compared to a specific reference point, an increase in respiratory health problems was observed [10]. Temperature fluctuations have been linked to changes in the composition of healthy nasal microbiota among young individuals, as demonstrated by Zhao et al. These alterations in microbiota can contribute to the development of respiratory diseases, particularly during transitional seasons like spring and fall when the weather is changing [44].

Hong-Ren Yu et al. found that a 1 °C temperature increase was a protective factor against asthma emergency room visits by adults and children. However, these weather conditions (temperature, atmospheric pressure, and humidity) coexist with air pollution so these should be considered when examining the effects of air pollution on acute exacerbation (AE) of asthma [46]. By inspecting the utilization of medical care data from a hospital emergency department, Jo et al. [39] found that lower mean temperature, lower relative humidity, large temperature difference, higher atmospheric pressure, and strong wind increased the utilization of medical care for asthma and COPD. Temperature was also seen as being responsible for altering healthy NP microbiota in the youth in a study by Zhao et al. [44]. Lower or colder temperatures are also associated with increased hospital visits due to allergic rhinitis, as shown in a study from Korea by Kim et al. [47]. Alvaro et al. found that a lower temperature is also associated with a higher risk of ALRI in children by providing higher stability to pathogens, causing hosts to be more susceptible to infections. The case is similar with higher humidity levels [40].

**Figure 3:**
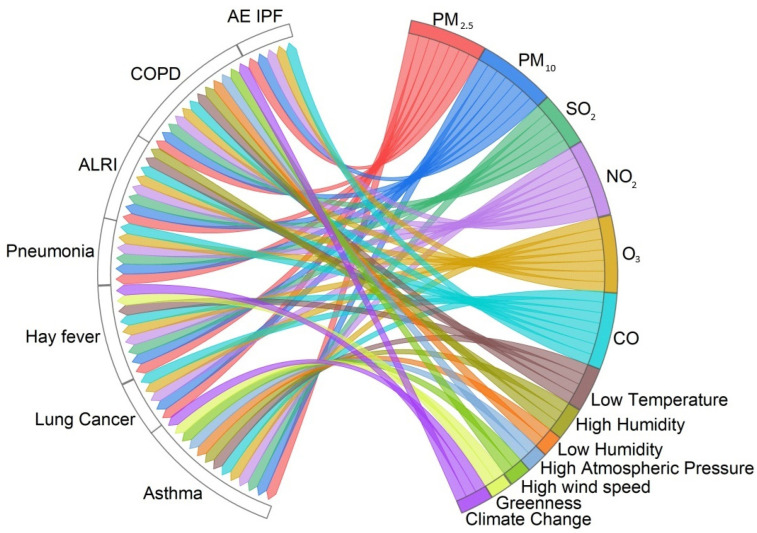
Depicts positive associations between respiratory health issues and environmental factors. (AE-IPF: acute exacerbation of idiopathic pulmonary fibrosis, COPD: chronic obstructive pulmonary disease, ALRI: acute lower respiratory infections, PM_2.5_: particulate matter smaller than 2.5 µg/m^3^, PM_10_: particulate matter smaller than 10 µg/m^3^, SO_2_: sulfur dioxide, NO_2_: nitrogen dioxide, O_3_: ozone, CO: carbon monoxide).

Figure 3 depicts a chord diagram showing positive associations between environmental factors (air pollution, weather conditions, greenness, and climate change) and respiratory health issues (asthma, lung cancer, hay fever, pneumonia, ALRI, COPD, and AE-IPF). The correlating chord diagrams presented in this scoping review were generated using the Origin Pro software. Each link in the chord diagrams shows a positive value association between the disease and the environmental factor as identified by reviewed articles. Also, in the case of the interplay of environmental factors with each other, links in the chord diagram show positive association values. It is important to note that the links in the chord diagrams show a positive association and are not quantified.

### 3.2. Cardiovascular Health

Environmental factors like air pollution and extreme temperatures, both hot and cold, have been observed to be positively correlated with cardiovascular health issues. Argacha et al. explained how air pollution control has become a necessity for the prevention of cardiovascular health issues [48]. Moreover, extreme temperatures, both hot and cold, can trigger a cardiovascular episode. In an umbrella review by De Bont et al. [49], we observed that air pollutants have strong links with various types of cardiovascular diseases (CVDs) like cardiac arrest, heart failure, stroke, and heart attack (myocardial infarction or ischemic heart disease). Whether the exposure to air pollutants is long-term or short-term, cardiovascular health issues increase. Moreover, Niu et al. reported positive associations between PM_2.5_, PM_10_, SO_2_, and NO_2_ and stroke incidence and mortality [50,51]. Liu et al. investigated cellular and molecular levels and found links between cardiovascular dysfunction and ambient PM_2.5_ exposure [52]. 

Crouse et al. [16] reported an increased risk of cardiovascular mortality due to increased long-term PM_2.5_ exposure. Likewise, a large-scale study of the literature by Riggs et al. [2] linked premature cardiovascular mortality to acute PM_2.5_ exposure and chronic cardiovascular events such as myocardial infarction, stroke, or arrhythmias. Pena et al. also discussed the association between exposure to air pollution and CVDs. They stated that chronic exposure to air pollution is a high-risk factor for many CVDs, such as high blood pressure, atherosclerosis, hypertrophic cardiomyopathy, angina, stroke, heart attack, heart failure hospitalization, arrhythmia, cardiac arrest, heart failure hospitalization, and cardiovascular mortality [53]. Furthermore, while investigating the exposome concept, which follows all the exposures of an individual from birth to death, air pollution was found to be the leading risk factor for cardiovascular diseases by Daiber et al. [54]. According to the Lancet Commission and The Global Burden of Disease, air pollution is now a well-known contributor to CVDs [54].

A 15-year-long study by Jalali et al. demonstrated how PM_2.5_ is an important risk factor for contracting CVDs [55]. The fine particles in PM_2.5_ can penetrate the respiratory system, from blood to other organs, and even the brain. The World Health Organization (WHO) advises against prolonged exposure to severe PM_2.5_ concentrations over 10–25 μg/m^3^ since this may increase inflammation, harm blood vessels, and, finally, result in cardiovascular disease. Hayes et al. highlighted the importance of studying the effects of PM_2.5_ exposure at lower levels due to its relevance in predicting future benefits and risks. However, the scarcity of research in such environments has led to an insufficient understanding of the relationship between air pollution and cardiovascular disease mortality [56]. 

According to the WHO, over 7 million people worldwide pass away every year because of breathing air pollution, with 3.3 million of those deaths being directly related to CVDs. The elderly, individuals with pre-existing CVDs, and children are particularly vulnerable. Kim et al. demonstrated the relationship between early life exposure to chronic air pollution and key CVD risk factors [57]. They discovered links between PM_2.5_, PM_10_, NO_2_, NO_x_, SO_2_, and O_3_ with stroke, and PM_2.5_ with atherosclerosis. They also discovered links between PM_2.5_, PM_10_, black carbon, NO_2_, CO, SO_2_, and O_3_ with cardiac arrhythmias. Wei Du et al. demonstrated that increased personal exposure to PM_2.5_ also increased hypertension risks [58]. Similarly, compared to exposures to filtered air, BP levels were higher throughout coarse PM exposure [59]. Similarly, Bo-Yi et al. suggested a link between elevated arterial BP and pre-existing hypertension and exposure to ambient air pollutants (PM_2.5_, PM_10_, NO_2_, NO, and SO_2_) [60].

Several epidemiological studies found daily ambient temperature changes to be correlated with cardiovascular mortality and morbidity. Ma et al. showed that every one-degree centigrade temperature increase or decrease is associated with cardiovascular health risk [10]. Khatri et al. found associations between multiple components of air pollution (e.g., elemental carbon, NO_2_, SO_2_, nickel, and vanadium) and cardiovascular or respiratory hospitalizations [31]. Furthermore, Aronson et al. researched associations between exacerbations of acute heart failure and increased respiratory infections in winter [61]. As investigated by Xuying et al., cardiovascular mortality increases during both extremely low and high temperatures [62]. On the other hand, Yeager et al. reported that greenness reduces the risk of CVD due to its ability to relieve stress and anxiety; it also decreases air pollution levels and promotes physical activity [63]. Argacha et al. reported that green spaces positively influence cardiovascular health [48]. Moreover, Miao Guo et al. found that relative humidity may be involved in developing atherosclerosis (thickening or hardening of arteries) and thrombosis (when blood clots block veins or arteries), which increases cardiovascular health issues in older people [64].

**Figure 4:**
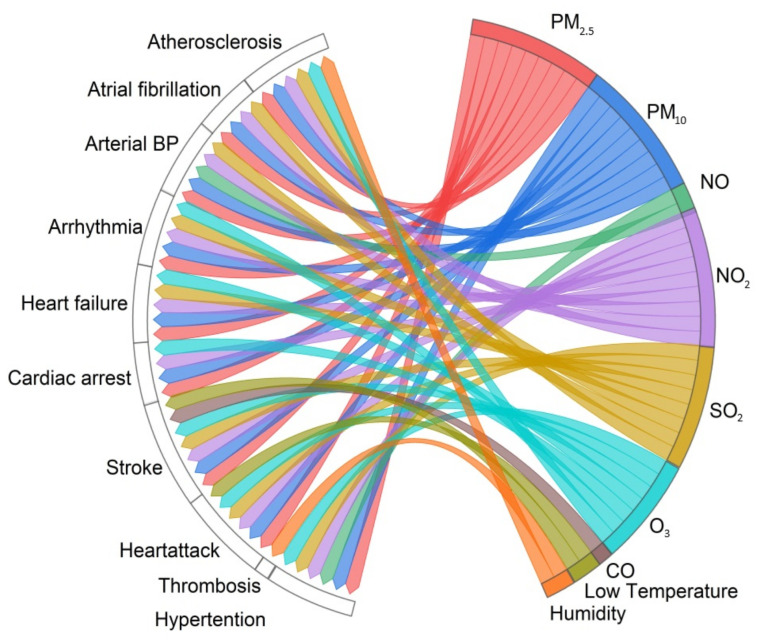
Depicts positive associations between cardiovascular health issues and environmental factors (PM_2.5_: particulate matter smaller than 2.5 µg/m^3^, PM_10_: particulate matter smaller than 10 µg/m^3^, SO_2_: sulfur dioxide, NO_2_: nitrogen dioxide, O_3_: ozone, CO: carbon monoxide).

In Figure 4, we show positive associations between environmental factors, including air pollution and temperature, with certain CVDs (atherosclerosis, atrial fibrillation, arrhythmia, cardiac arrest, heart failure, stroke, heart attack, and hypertension) using a chord diagram.

### 3.3. Metabolic and Gastrointestinal Health

Irritable bowel syndrome (IBS) is a multifactorial gastrointestinal disorder affecting 5.2–22% of the population [65]. Loo et al. found a link between allergic diseases such as asthma, allergic rhinitis (AR), atopic dermatitis (AD), and IBS. This means that when the air quality worsens, it causes not only allergies but IBS as well [65]. In Taiwan, it was seen that increased levels of CO, non-methane hydrocarbons (NMHC), NO_2_, and CH_4_ caused an increase in the incidence of IBS. It is known that the gut microbiome composition is altered in patients with IBS and allergic diseases. Due to this, increased IBS-dominated diarrhea is observed compared to healthy patients. Similarly, patients with asthma or allergies suffer more from gut microbiome alterations compared to healthy people. This demonstrates that changes in gut microbiota may later contribute to the development of IBS and allergic diseases. Similarly, Gu et al. found evidence of air pollution exposure adversely affecting certain diseases of the digestive system [66]. They observed increased hospitalizations for intestinal infections, esophageal disorders, gastritis or duodenitis, appendiceal conditions, liver diseases, gastrointestinal hemorrhage, and noninfectious gastroenteritis due to increased PM_2.5_ levels. 

PM_2.5_ exposure was also seen to be associated with acute appendicitis, inflammatory bowel disease, and peptic ulcer bleeding. Wu et al. found that short-term exposure to air pollution, including to SO_2_, NO_2_, CO, O_3_, and PM_2.5_, was significantly associated with increased hospitalizations with peptic ulcers, mostly at low temperatures [67]. PM_2.5_ and CO were identified by Ho et al. as risk factors for aggravating gastroesophageal reflux disease (GERD) [68]. Hamid et al. identified gastric cancer risk factors; these included drinking water, soil, food, and geographical conditions [69]. Beyond the composition of the foods themselves, factors such as air pollution, chemical contaminants in food and water, and early-life environmental exposures are being studied for their potential influences on the onset of food allergies [70].

There are also several other important endocrine health issues, such as diabetes, that are associated with both relatively low and high temperatures. Diabetes patients are at a greater risk of heat-related illnesses [71]. The evidence for the harmful effects of exposure to ambient air pollutants (especially PM) on type 2 diabetes and the evidence that diabetic patients may be more susceptible to air pollutant exposure has been strengthened by recent publications [72]. Similarly, Yaguang Wei et al. showed positive associations between PM_2.5_ and increased hospital admissions due to diabetes, renal failure, and other cardiovascular and respiratory diseases [73]. Yang et al. found that participants who lived in greener neighborhoods had a lower risk of diabetes mellitus and that higher levels of residential greenness may be associated with lower levels of diabetes prevalence and glucose homeostasis markers [74]. Hui-Ju et al. found that there is an inverse relationship between exposure to surrounding greenness and the incidence of type 2 diabetes after adjustment for individual-level covariates, comorbidities, and region-level covariates [17]. An interesting study by O’Donovan et al. [75] found a positive association between air pollution (NO_2_ and PM_2.5_) and type 2 diabetes, but the association appeared to depend upon demographic factors. Gu et al. also found positive associations between PM_2.5_ and diabetes mellitus [66].

**Figure 5:**
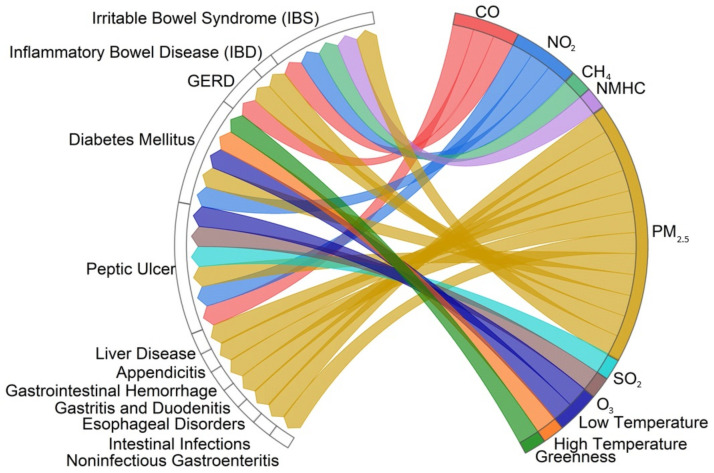
Depicts positive associations between metabolic and gastrointestinal health issues and environmental factors (GERD: gastroesophageal reflux disease, NMHC: non-methane hydrocarbons, CH_4_: methane, PM_2.5_: particulate matter smaller than 2.5 µg/m^3^, SO_2_: sulfur dioxide, NO_2_: nitrogen dioxide, O_3_: ozone, CO: carbon monoxide).

Figure 5 depicts the associations between certain metabolic and gastrointestinal health issues (IBS, peptic ulcer, noninfectious gastroenteritis, gastrointestinal hemorrhage, liver disease, appendicitis, gastritis or duodenitis, diabetes mellitus, IBD, gastroesophageal reflux disease (GERD), esophageal disorders, and intestinal infections) and air pollution, temperature, and greenness.

### 3.4. Renal and Urogenital Health

High outdoor temperatures and daily emergency ambulance dispatches for renal colic may be correlated nonlinearly and weakly, according to Yang et al. [76]. PM exposure can induce oxidative stress and inflammation, negatively affect glucose tolerance, and induce insulin resistance, particularly in the liver. It also has toxic effects on the pancreas and gastrointestinal system [11]. The kidneys process about 180 L of blood daily, making them susceptible to harm from exposure to PM, specifically PM_2.5_. Research indicates that exposure to this particulate matter is associated with a faster decline in glomerular filtration rate, reduced kidney function during pregnancy, heightened risks of chronic kidney disease (CKD), end-stage renal disease (ESRD), kidney failure, and an increase in mortality related to chronic kidney disease. CKD is described as a condition with reduced renal function, characterized by a glomerular filtration rate (GFR) below 60 mL/min per 1.73 m^2^, or markers of renal impairment lasting for at least 3 months. ESKD is considered the stage of CKD where the GFR drops below 15 mL/min per 1.73 m^2^, and renal function is no longer sustainable for life without treatments such as dialysis or renal transplantation [77]. 

A study by Shih-Yi et al. investigated the association between air pollutants and the risk of chronic kidney disease (CKD) and end-stage renal disease (ESRD) in Taiwan. They found that higher concentrations of SO_2_, NO_x_, NO, NO_2_, and PM_2.5_ were linked to an increased risk of CKD and ESRD [18]. However, in Korea, Seo Yun et al. found that only CO exposure significantly increased CKD risk in individuals under 60 years of age [78]. Hongyan Liu et al. also found similar results indicating that long-term exposure to PM_2.5_, PM_10_, CO, NO_x_, SO_2_, and O_3_ increased the risk of CKD [79]. Sidar et al. also discussed the potential impact of air pollution particularly of PM_2.5_ on the CKD burden [19,80]. A study by Chih Da et al. found positive links between PM_2.5_ and ESRD and identified an inverse association between the incidence of ESRD and greenspaces [81]. Hui-Ju et al. also discussed the link between various environmental pollutants (PM and heavy metals) and CKD, emphasizing the need to understand this association to address CKD as a global public health issue [17].

**Figure 6:**
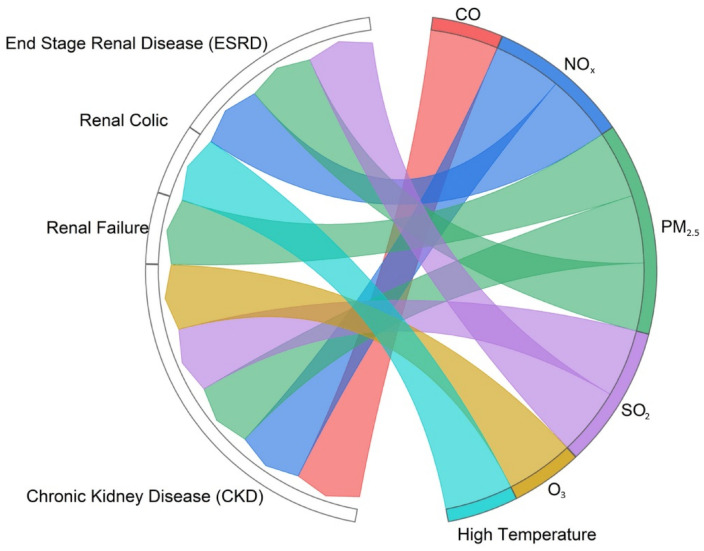
Depicts positive associations between renal and urogenital health issues and environmental factors (PM_2.5_: particulate matter smaller than 2.5 µg/m^3^, SO_2_: sulfur dioxide, NO_x_: nitrogen oxides, O_3_: ozone, CO: carbon monoxide).

Figure 6 shows the associations between certain renal and urogenital health issues (renal failure, renal colic, chronic kidney disease (CKD), and end-stage renal disease (ESRD)) and air pollution and temperature.

### 3.5. Neurological and Psychological Health

Since mental health is an inseparable aspect of modern human health, global concerns about mental health are on the rise, and it is believed that a variety of factors, including demographic factors like socioeconomic status and health behaviors along with ecological environmental factors, affect mental health conditions. The literature contains much evidence that environmental factors such as green spaces and PM levels are associated with mental health. While exposure to green spaces can have a positive effect on mental health, PM pollution can cause inflammation in the central nervous system, which causes mental health problems. Kim et al. discovered that increasing community access to greenness and decreasing levels of PM are positive and crucial components in improving mental health conditions among urban inhabitants [82]. Parks and urban forests provide locals with places to interact and engage in outdoor activities, which improves their mental health. The sense of social satisfaction and overall happiness of locals can be increased by a greener environment. In populated urban areas, these findings are more reliable [83].

Gu et al. reported that air pollution significantly causes psychological disorders such as depression, feeling nervous, powerless, and restless. They further found that air pollution affects mental health both directly by causing inflammation in the nervous system and indirectly by mitigating general well-being [84]. Today, anxiety disorders are among the most prevalent mental health issues. A Chinese study by Chen et al. [85] found a very significant association between PM_2.5_ exposure and anxiety disorders. They found an increase in anxiety disorders for every unit of increase in PM_2.5_ level. Similarly, He et al. [86] examined the possible association between PM_2.5_ and depression and found that a 10 μg/m^3^ increase in the previous week’s PM_2.5_ was significantly associated with an increase in depression levels. This phenomenon is more prominent in the spring and summer. PM_2.5_ also correlated dangerously with suicide ideation, as Chen et al. found in a Chinese study [87]. 

The literature also showed that children, specifically adolescents, are at risk for mental disorders, with, in 2019, 1200 disability-adjusted life years (DALYs) per 100,000 people with depressive disorders. These risks grow with age. In addition, Turner et al. explored the effects of PM_0.1_ on adolescent health. They found that PM exposure adversely affects the development of the central nervous system and brain [88]. PM_0.1_ exposure has been found to cause decreased intelligence and memory, self-reported anxiety and depression, and increased pediatric psychiatric emergency department utilization. PM_0.1_ was also associated with anxiety, depression, and physical stress. PM was also found to be responsible for neuro-inflammation and neurological dysfunction because it can cross the blood–brain barrier [89]. Cruz et al. suggested there is an association between green spaces with lakes and the frequency of serious mental illnesses such as schizophrenia, bipolar affective disorder, or psychosis [90].

Xue et al. found that people with mental disorders such as schizophrenia find it difficult to maintain their body temperatures when they are exposed to high temperature fluctuations, suggesting ambient temperature changes have negative effects on people’s mental disorders [89]. In Guangzhou, China, Gao et al., while assessing physical and mental health, found that thermal environmental factors are more impactful on physical and mental health than atmospheric environmental factors and that physical health is more affected by environmental factors than mental health [91]. In an Italian study by Aguglia et al. [92], results showed significant associations between temperature fluctuations and humidity and emergency psychiatric ward visits. The literature showed that higher temperatures are very likely to cause psychiatric disorders, increased hospital visits or admissions to emergency psychiatry wards, and death. Similarly, hospital emergency room visits with mood disorders, behavior disorders, neurotic disorders, and schizophrenia also increase due to high temperatures [93].

Palinkas et al. investigated the direct and indirect impacts of climate change on mental health. These impacts can be acute (anxiety, mood disorders, acute stress reactions, posttraumatic stress disorders, sleep disruption, and suicide), sub-acute (aggressive or criminal behavior, lethargy, low mood, and cognitive impairment), and long-term (psychological distress, anxiety about the future, eco-anxiety, despair, and hopelessness) [94]. Luong et al. found that poor mental health persists even after a drought and found the relationship to be an inverted u-shaped pattern, which shows that people experience an increase in stress in the first few years of drought, and, after some time, distress dissipates but life satisfaction decreases overall [95]. Extreme climate or weather events and environmental quality decline also affect the mental health of people living in rural areas. Batterham et al. attempted to find associations between environmental factors such as drought, climate, extreme weather events, and green spaces and mental health and concluded that extreme climate or weather events have a negative impact on mental health in rural areas [96]. PM exposure is associated with an increased risk of neurological diseases, including Alzheimer’s, Parkinson’s, multiple sclerosis, dementia, and autism spectrum disorder [11].

**Figure 7:**
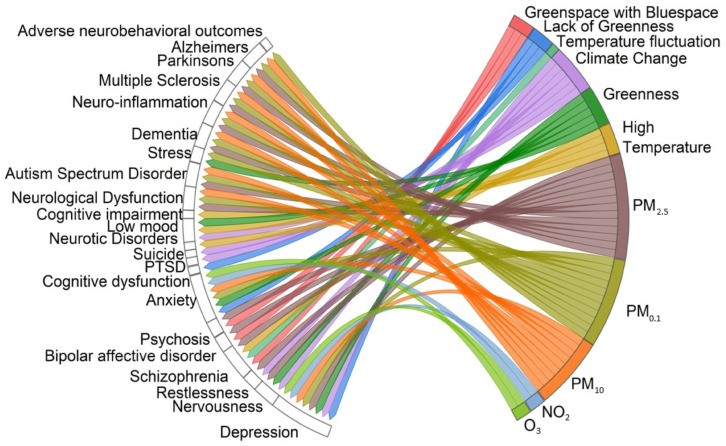
Depicts positive associations between neurological and psychological health issues and environmental factors (PTSD: Post-Traumatic Stress Disorder, PM_2.5_: particulate matter smaller than 2.5 µg/m^3^, PM_10_: particulate matter smaller than 10 µg/m^3^, PM_0.1_: particulate matter smaller than 0.1 µg/m^3^, NO_2_: nitrogen dioxide, O_3_: ozone).

The chord diagram (Figure 7) illustrates the associations found between neurological and psychological health issues (anxiety, depression, nervousness, restlessness, suicide, schizophrenia, bipolar affective disorder, psychosis, PTSD, cognitive dysfunction, low mood, neuro-inflammation, neurotic disorder, stress, cognitive impairment, Alzheimer’s, Parkinson’s, Multiple Sclerosis, dementia, autism spectrum disorder, and adverse neurobehavioral outcomes) and air pollution, climate change, greenness, and weather conditions.

### 3.6. Infectious Diseases

Over 70% of infectious diseases are caused by the environment we live in. Moreover, more than half of human pathogenic diseases are a result of climate change [97]. This makes the natural environment a key contributor to fatal/infectious human health issues and a review of the literature investigating these issues is justified. Wang et al. found that humidity was negatively associated with dengue fever and the incidence of measles disease was inversely correlated with monthly precipitation but positively correlated with monthly wind speed [32,98]. Both the average air temperature and the amount of precipitation had a significant impact on the incidence of chickenpox [32,99]. In addition, a rise in temperature, relative humidity, and duration of sunshine may increase the risk of malaria infection [100]. Dengue fever is a climate-responsive disease and temperature is regarded as one of the most important climatic factors for dengue transmission [101]. Similarly, daily vapor pressure and mean and minimum temperatures were associated with increased dengue fever risk [102]. Kari et al. demonstrated that a decrease rather than low temperature and humidity per se during the preceding three days increases the risk of influenza episodes in a cold climate [103].

The COVID-19 pandemic scenario demonstrated how various environmental factors, such as air pollutants and meteorological conditions, could influence how a contagious disease affects human health. Coronavirus is a temperature-sensitive virus [76]. Certain weather conditions influence virus transmission rates, while air pollution affects respiratory health and potentially exacerbates the effects of diseases such as COVID-19. In essence, there is a complex interplay between environmental factors and disease transmission, and COVID-19 pandemic studies underscore the importance of understanding how environmental conditions can impact the course and severity of infectious diseases [20,25,28,104]. A recent study conducted in the United States (USA) by Fang et al. suggested that exposure to PM_2.5_ and PM_10_ increases the risk of COVID-19 infection [105]. They also revealed that long-term exposure to PM_2.5_ increases the risk of COVID-19 mortality. Another study by Zoran et al. in Italy determined that PM_10_ had the strongest link with the number of COVID-19-related deaths [104].

**Figure 8:**
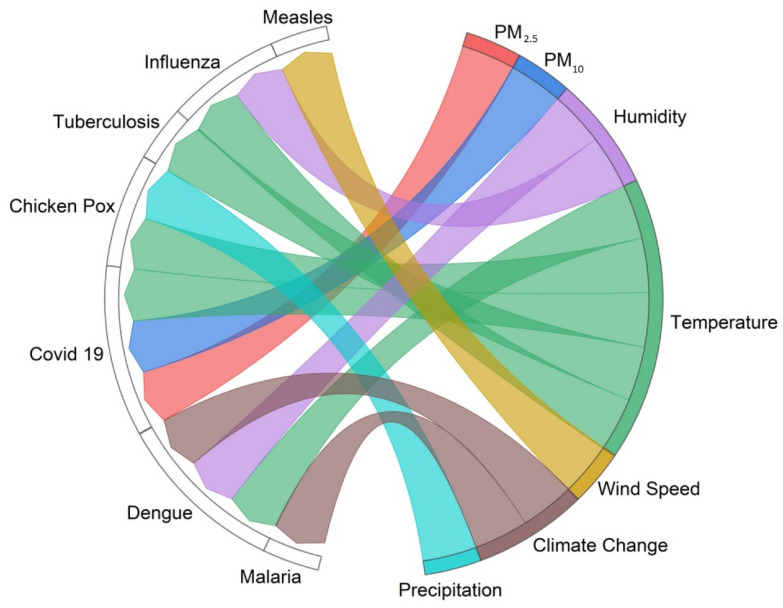
Depicts positive associations between infectious diseases and environmental factors (PM_2.5_: particulate matter smaller than 2.5 µg/m^3^, PM_10_: particulate matter smaller than 10 µg/m^3^).

Kai et al. found that exposure to low temperatures is associated with an acute increased risk of tuberculosis hospitalizations and the association between temperature and tuberculosis admission varies depending on air pollution, latitude, and medical resources [106]. We have shown the associations between certain infectious diseases (malaria, dengue, measles, chicken pox, COVID-19, tuberculosis) and air pollution, climate change, weather conditions, and greenness using a chord diagram (Figure 8).

### 3.7. Oncological Diseases

Cancer is one of the major fatal health issues, and, certainly, many environmental factors aid in its incidence, mortality, and morbidity. Here, we discuss some of the most common environmental factors whose associations with cancer were discovered in the reviewed literature. Compared to other environmental factors, a country’s average annual temperature may significantly contribute to cancer deaths, according to a multivariate analysis of 188 different countries by Sharma et al. [107]. According to this study’s findings, people who live in cold climates may be at an increased risk of developing cancer and dying from it. The reason could be that there is more ultraviolet B radiation exposure near the equator and UVB exposure helps to synthesize vitamin D, which in turn helps prevent cancer. There are few food sources of vitamin D, and exposure to UVB radiation from sunshine is the primary way of absorbing it. Cloud cover, stratospheric ozone, height above sea level, skin pigmentation, the number of hours spent inside, and the type of clothing all have an impact on sun exposure. These studies also suggested that there might be factors other than vitamin D and UVB that help prevent cancer mortality and incidence, but they might be related to a specific geographical location. The same phenomenon is also explained by Lucas et al. [108].

Purushothaman et al. showed the association between chronic vitamin D deficiency and colorectal cancer incidence and how it increases with age. They also suggested improving UVB exposure guidelines for better absorption of vitamin D depending on specific geographical areas [109]. Similarly, Berman et al. highlighted the geographic and environmental variations in Canada that are associated with skin cancer. They pointed out that an increase in annual average temperature, summer UVR, and green spaces is associated with an increase in the incidence of skin cancer, whereas an increase in the number of annual heat events combined with the highest annual temperature and an increase in the average number of annual rain events are associated with a decrease in the incidence rate of skin cancer [110].

Najafi et al. showed how solar UV radiation has associations with the incidence of gastrointestinal cancers such as esophageal, stomach, and colon cancers [111]. They showed that geographical locations with higher UV index levels have a relatively low incidence of gastrointestinal cancers. UV rays might serve as a preventative measure against certain gastrointestinal cancers. In the literature, the geographical location of cities as well as exposure to sunlight may be protective factors against these cancers. The observation of more cancer deaths at high latitudes has led to the hypothesis that sunlight may prevent some cancers and that obtaining vitamin D reduces cancer risk. Therefore, there is a link between latitude and esophageal, stomach, and colon cancers as well as several other cancers. Haidari et al. [112] have also reported that in addition to standard cancer treatment, sun exposure and/or vitamin D supplementation can reduce the risk of colorectal cancer.

Another study by Kiani et al. [113] from Iran used a geographical information system-based approach to assess different cancers and how environmental factors are related to them. Their results showed associations between colon cancer and exposure to concentrations of heavy metals such as cobalt. The purpose of a Brazilian study by Yu et al. [114] was to determine the relationship between long-term exposure to PM_2.5_ and cancer-specific mortality. Long-term PM_2.5_ exposure was found to be strongly associated with various cancer-specific mortalities, including oral, nasopharynx, esophageal, stomach, colon, rectal, liver, gallbladder, larynx, lung, bone, skin, female breast, cervix, prostate, and brain cancers and leukemia. They showed that cancer mortality was significantly impacted by even very low PM_2.5_ concentrations. The consumption of inhaled tobacco and environmental pollution are the known main culprits of lung cancer, but Sapunar et al. adjusted the model for smoking and still found a significant direct association between high concentrations of PM_2.5_ and PM_10_ and the incidence of adenocarcinoma (lung cancer) [115].

Likewise, Gharibvand et al. [116] conducted a study in a cohort of non-smokers and revealed that the risk of adenocarcinoma rose by 31% for every 10 μg/m^3^ rise in the concentration of PM_2.5_. According to Gariazzo et al. [117], inhaling tiny particulate matter (PM_2.5_) increases the probability of dying from lung cancer from 12.10% to 14.96% for every additional 10 μg/m^3^. Additionally, PM_2.5_ is responsible for 14.05% of male lung cancer cases and 16.26% of female lung cancer cases. It is estimated that in 2030, there will be three million new cases of breast cancer worldwide [118] and 900,000 deaths from this cause, according to the World Health Organization’s predictions. In Chile, Zenteno et al. found a direct link between NO_2_ and the incidence of breast cancer [118]. Similar results are found in other geographical locations such as Canada and the US. Findings from Giannoula et al. suggested that benzo (k) levels, a PAH (polycyclic aromatic hydrocarbon), are associated with the occurrence of thyroid cancer [119]. PAHs are also linked to an increased risk of lung and skin cancers.

**Figure 9:**
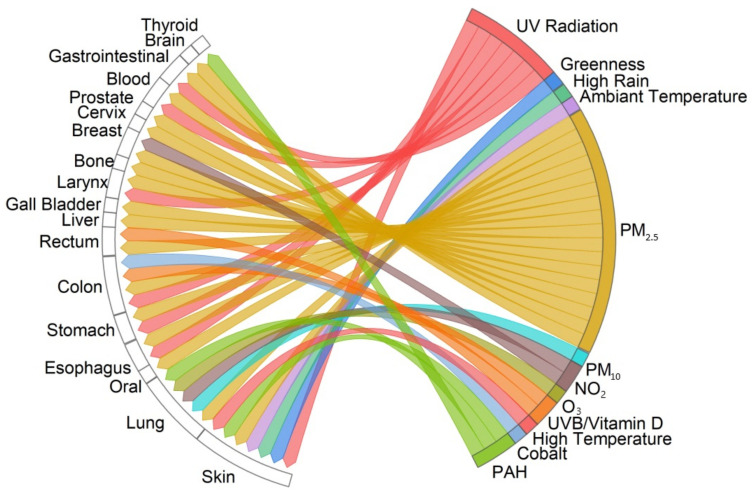
Correlates Cancer with environmental factors (PM_2.5_: particulate matter smaller than 2.5 µg/m^3^, PM_10_: particulate matter smaller than 10 µg/m^3^, NO_2_: nitrogen dioxide, O_3_: ozone, UVB: ultraviolet B radiation, PAH: polycyclic aromatic hydrocarbon).

The chord diagram (Figure 9) shows associations between air pollution, solar radiation, greenness, and weather conditions and certain types of cancers (skin, lung, oral, esophagus stomach, colon, rectal, liver, gall bladder, larynx, bone, breast, cervix, prostate, blood, gastrointestinal, and brain). These associations were drawn from studies that identified a positive correlation between environmental factors and the cancer type.

### 3.8. Skin Diseases

Several environmental stressors target the skin at its interface with the air. Angioedema is the swelling of the deeper layers of the skin and is caused by a buildup of fluid. It is frequently the result of an allergic reaction and can lead to potentially fatal complications. According to Kedarisetty et al., higher levels of air pollution, specifically ground-level O_3_, are associated with significantly higher rates of angioedema episodes [120]. This could be due to lower levels of air pollution around the greenspace, increased physical activity in the greenspace, or decreased adiposity. Patella et al. showed that weather variations and air pollution have a substantial impact on the symptoms and skin responsiveness of atopic dermatitis (AD) patients, worsening their dermatitis. AD was also found to be associated with DTR 14 °C, relative humidity, PM_10_, and NO_2_ [121]. Wang et al. found associations between hydrocarbons such as CH_4_ and atopic dermatitis, also named atopic eczema [32]. Moreover, Anke Huls et al. found that exposure to certain air pollutants, such as NO_x_, PM_2.5_, and PM_10_ is associated with an increased risk of eczema, particularly non-atopic eczema [122].

Dai Miyazaki et al. highlighted the increasing prevalence of allergic conjunctivitis worldwide based on data from global surveys and population-based studies [123]. They demonstrated the effects of pollen dispersion, climate, and air pollution (NO_2_, PM_10_, PM_2.5_, O_3_, SO_2_, and traffic-related air pollution (TRAP)) on the prevalence of allergic conjunctivitis. PM exposure can cause oxidative stress, mitochondrial dysfunction, and altered gene expression related to skin barrier function and immune response, leading to skin damage. Irini M. Dijkhoff et al. discussed the detrimental effects of PM on human skin, including the exacerbation of skin diseases such as atopic eczema and the acceleration of skin aging. They also emphasized the role of other environmental stressors, such as ozone and UV radiation, in skin health and the importance of considering their combined effects with PM [124].

There is a link between exposure to air pollutants and the development or exacerbation of skin diseases. Ruhollah Abolhasani et al. reviewed the evidence linking air pollution to conditions such as skin aging, atopic dermatitis, cellulitis, psoriasis, and skin cancer. Exposure to pollutants like PM_10_ and NO_2_ during pregnancy and early childhood is associated with an increased risk of AD and childhood eczema. In addition, these pollutants cause skin damage, including oxidative stress, inflammation, and skin barrier dysfunction [125]. E. Araviiskaia et al. highlighted the interactions between pollutants (PM, O_3_, NO_x_, and SO_2_) and UV radiation and the potential clinical consequences, like chronic inflammatory skin diseases in people living in a polluted environment [126].

**Figure 10:**
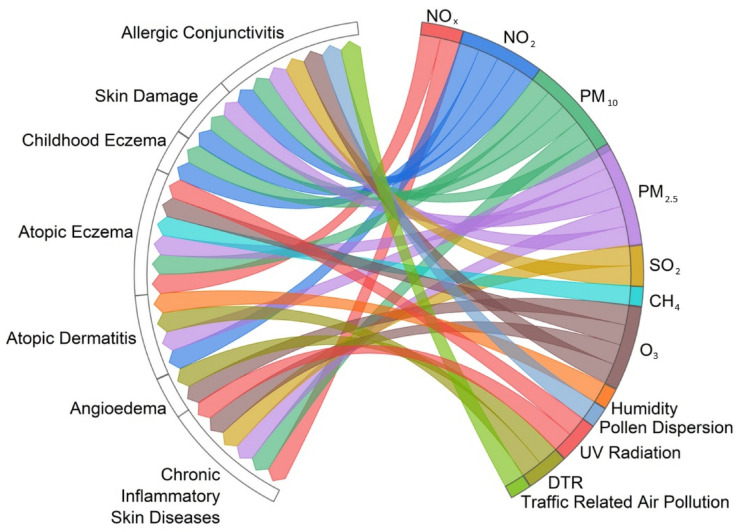
Correlates skin diseases with environmental factors (PM_2.5_: particulate matter smaller than 2.5 µg/m^3^, PM_10_: particulate matter smaller than 10 µg/m^3^, NO_2_: nitrogen dioxide, NO_x_: nitrogen oxides, O_3_: ozone, UV: ultraviolet radiation, DTR: diurnal temperature range).

The chord diagram (Figure 10) shows associations between air pollution, solar radiation, and weather conditions and certain types of skin diseases (angioedema, atopic dermatitis, atopic eczema, allergic conjunctivitis, skin damage, childhood eczema, and chronic inflammatory skin diseases).

### 3.9. Other/Miscellaneous Diseases

Yue Chen et al. focused on the relationship between air pollution exposure and the risk of outpatient visits with Sjogren’s syndrome (SS) in Hefei, China. Their findings indicated that exposure to PM_2.5_ and NO_2_ was associated with an increased risk of outpatient visits with SS [127]. Similarly, Tian-Ping et al. reported that exposure to NO_2_, SO_2_, O_3_, and CO was associated with an increased risk of SS hospitalization [128]. Rheumatoid arthritis (RA) is a chronic disorder of inflammation that targets joints and cartilage and leads to severe disability. Jiyoung Shin et al. found a positive association between exposure to O_3_ and CO and the risk of developing RA in adults over 20 years of age [129]. Similarly, Wen-Chao Ho et al. in Taiwan found that exposure to PM_2.5_ is associated with an increased risk of RA [130]. Marina et al. reported that studies related to the environment and RA have been increasing and increased RA risk is linked with silica, CO, O_3,_ and other eco-toxicants [131]. Xiaoyu Xi et al. found an association between polycyclic aromatic hydrocarbons (PAHs) and RA [132].

Chronic sinusitis (CS) is a multifactorial inflammatory condition in the paranasal sinus mucosa. Mengxue Lu et al. found that exposure to outdoor air pollutants, particularly PM_2.5_ and NO_2_, was associated with an increase in outpatient CS cases, especially among the youth [133]. A review by Evelyn M. Leland et al. found that air pollution, particularly PM, is correlated with the incidence/prevalence and severity of chronic rhino sinusitis [134]. Jelle Vehof et al. investigated the prevalence and risk factors of dry eye in the Netherlands. They found that NO_2_ was significantly associated with dry eye, and this association may be partly due to an increased prevalence of other diseases linked to air pollution [135]. M. Volkan Akdoğan et al. investigated the relationship between meteorological factors, air pollution, and the frequency of epistaxis (nosebleeds) in children. Their findings revealed that high temperatures were positively correlated with epistaxis rates, while humidity, particulate matter, sulfur dioxide levels, air pressure, and rainfall showed a negative association [136].

Agne et al. reviewed articles that identified specific air pollutants, such as PM_2.5_, PM_10_, CO, NO_2_, O_3_, and SO_2_, associated with an increased risk of eye diseases. Significant associations were found between PM_2.5_ and glaucoma, PM_2.5_ and PM_10_ were found to be associated with diabetic retinopathy, and CO, NO_2_, and PM_10_ were found to be linked to an increased risk of central retinal artery occlusion [137]. Amy E. Millen et al. also found consistent associations, suggesting a possibly increased risk of cataract and retina-associated chronic eye disease with increasing exposure to PM_2.5_, PM_10_, NO_2_, NO_x_, and SO_2_ [138]. Moreover, Wanzhou Wang et al. found that short-term exposure to PM_2.5_, PM_10_, CO, and NO_2_ is linked to increased visits for eye diseases and conjunctivitis. The effects of air pollutants on eye diseases are also influenced by temperature, with stronger effects observed in extreme temperature conditions. They emphasized the importance of controlling air pollution, particularly on days with higher or colder temperatures, to protect eye health [139]. Similarly, Si-Yu Gui et al. showed that extreme weather conditions, such as low temperatures and high humidity, increase the risk of dry eye disease visits. However, high atmospheric pressure reduces the risk [140].

**Figure 11:**
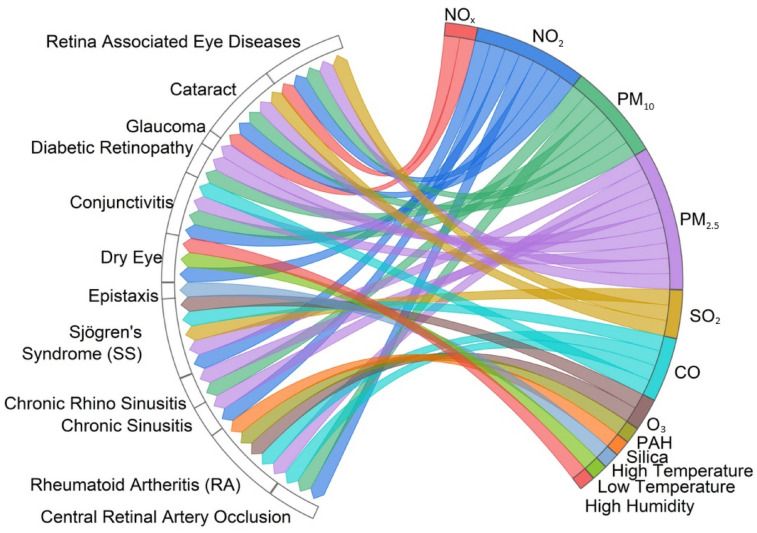
Correlates other/miscellaneous diseases with environmental factors (PM_2.5_: particulate matter smaller than 2.5 µg/m^3^, PM_10_: particulate matter smaller than 10 µg/m^3^, NO_2_: nitrogen dioxide, NO_x_: nitrogen oxides, O_3_: ozone, CO: carbon monoxide, SO_2_: sulfur dioxide, PAH: Polycyclic Aromatic Hydrocarbon).

The chord diagram (Figure 11) shows associations between air pollution and weather conditions with certain diseases related to rheumatology, otolaryngology, and ophthalmology (Sjögren’s Syndrome, rheumatoid arthritis, chronic sinusitis, chronic rhino sinusitis, dry eye, epistaxis, glaucoma, diabetic neuropathy, central retinal artery occlusion, cataract, retina associated chronic eye diseases, and conjunctivitis).

### 3.10. Climate Change

Because of the influence of human behavior, the climate is changing radically. Extreme and moderate weather events such as rising temperatures, reduced air quality, and climate change affect physical and mental health. Therefore, we must include its associations when we review general human health. There is a great deal of evidence in the literature for mortality and morbidity due to high ambient temperature. Droughts have affected over 2 billion people in the last two decades and can also introduce high PM levels and strong winds, which increase respiratory disease burden. Floods and wildfires also increase post-traumatic stress disorder (PTSD), depression, and anxiety. Wildfires increase air pollution levels and are associated with respiratory problems.

Climate change and the hazards related to it are associated with long-term mental disorders such as anxiety and grief and experiencing stress about climate change, which is now called climate anxiety or eco-anxiety. Extreme weather and climate events will become more frequent and more intense over the next decades, potentially having a large impact on people and healthcare systems throughout the world. Shi-Zhou et al. explained the direct and indirect impacts of climate change on allergic respiratory diseases and illustrated the combined effects of heat, air pollution, and aeroallergens that cause excess mortality and hospital admissions for allergic respiratory diseases [141].

In [142], Ebi et al. suggested the development of a better climate-resilient healthcare system along with enhanced climate predictions and alerts since climate change has become an extreme risk factor for global mortality, morbidity, and mental health decline. With an average of 60,000 deaths every year over the past 10 years, or 0.1% of all worldwide mortality, disaster-related deaths have a significant yearly fluctuation. In 2019, there were 396 disasters affecting millions across the globe both directly and indirectly, and this number is projected to increase every year as climate variability increases. Disasters like cyclones or hurricanes are highly detrimental to human health and correlate with death, diseases, infections, and mental health impacts.

Climate change may have a greater impact on some regions and populations than others. In [143], reviewing climate change effects, Clayton et al. classified climate change effects into four categories. The first category of effects are distinct events. They include floods, droughts, wildfires, and hurricanes, and are found to be associated with long-term and short-term mental health issues like depression, anxiety, PTSD, and even suicide. The second category of climate change effects are also immediate consequences from slower changes such as rising sea levels, warming temperatures, and changed precipitation patterns. Gradual changes in the environment also place psychological pressure on people. The third category of climate change effects is the way climate change affects physical and social systems; these have indirect impacts on mental well-being. The fourth category of climate change effect is the consequences of how people perceive climate change.

Liu et al. [144] showed that two-thirds of the population had PTSD stress due to a hurricane in 2017. Increasing temperatures and droughts have caused increased levels of anxiety and depression, which leads to suicide and suicidal thoughts. While reviewing the evidence of climate change-related environmental factors such as temperature, humidity, rainfall, and extreme events on respiratory allergic diseases and respiratory infections in children, Di et al. explained that there is a harmful interaction between climate change, temperature, pollutants, and pollen [145]. Significant associations were found between extreme temperatures, temperature with asthma, cold, and flu, allergic rhinitis, and relative humidity in patients with asthma. Thunderstorm-associated asthma resulting from extreme climate change events is related to preceding days of extreme grass pollen and strong winds.

While assessing the health impacts of climate change in Europe, Wilnhammer et al. found evidence of adverse mental health, and cardiovascular, respiratory, and all-cause mortality due to heatwaves, cold stress, droughts, wildfires, and floods [146]. They found that short-term heatwaves increased air pollution concentrations of PM, O_3_, NO_2_, and SO_2_ which, in turn, when inhaled, caused damage to the respiratory system. Floods have associations with long-term mental disorders such as PTSD and anxiety, along with several infectious diseases like dengue and malaria.

Di et al. demonstrated that climate change appears to be closely linked to air pollution. NO_x_, CO, and O_3_ are produced more quickly when the temperature is high. On the other hand, climate change-induced variations in temperature, precipitation frequency, and forest fires are likely to cause an increase in the concentrations of pollutants such as fine PM, PAHs, and black carbon [145]. It is thought that high temperatures brought on by global warming will affect the time of the year that plant pollen production begins and how long it lasts. The most studied locations have experienced a large rise in pollen load and length of the pollen season over time, which is correlated with yearly cumulative temperature rises.

### 3.11. Associations Between Environmental Factors That Affect Human Health

Another very important phenomenon to consider when investigating associations between environmental factors and health issues is the association between these environmental factors. All these environmental factors coexist and, therefore, affect each other directly and then later have adverse effects on human health as well. In a study by Alvaro et al., it was observed that temperature affects the movement of air pollution particles and has a positive association with PM_10_ levels [40]. Moreover, cold weather increases NO_2_, SO_2_, PM_10_, and CO levels, hot weather promotes the emergence of O_3_, while humidity decreases O_3_ pollution. Another possible link was found between ultraviolet radiation and PM_10_ in specific countries, indicating that ultraviolet radiation was attenuating the effect caused by PM_10_.

An interesting association was found between PM_10_ and air temperature by Ma et al. [10]. They reported a 10 μg/m^3^ increase in PM_10_ concentrations when the air temperature was relatively high. This resulted in a 0.92% increase in cardiovascular mortality. They discovered that NO_2_ and PM_10_ were positively correlated with each other while demonstrating inverse and weak links with ozone levels. Wu et al. discussed the joint effects of air pollution and temperature on hay fever [42]. It was seen that air pollution caused hay fever at lower temperatures. Weather conditions affect air pollutants, as reported by Alvaro et al. [40]. In addition, ozone in the troposphere, a dangerous photochemical pollutant, is amplified on warm, sunny days [147]. In addition, the lack of reliable data for spatial interpolation of daily air pollution in certain areas, particularly rural communities, presents a significant challenge. These areas often lack the necessary environmental monitoring infrastructure to measure air pollutants accurately. This gap in data is critical because while some studies have indicated that air pollutants may confound the temperature–mortality relationship, other studies have not found a significant confounding effect. This inconsistency suggests that more precise local-level data on air pollution are essential for clearer insights [120].

By investigating the relationship between greenness, air pollution, and mortality, Crouse et al. [16] found that there was no risk of increased mortality due to increased long-term exposure to PM_2.5_ in people living in greener residential areas compared to people living in areas with less residential greenery. This suggests that studies correlating air pollution with mortality must also consider including greenness as an important factor. Lefler et al. suggested that associations among air pollutants also make it difficult to access independent associations between these air pollutants and mortality. For example, the association between PM_2.5_ and NO_2_ likely contributed to instability in the estimated effect of NO_2_ exposure [26]. This underscores that there is difficulty in identifying and interpreting the specific health effects attributable to individual pollutants in the presence of multiple, interrelated environmental factors. In [42], Wu et al. discovered that it was difficult to separate the effect of each modifier on air pollution in a single city like Beijing because of the overlapping effect of temperature and humidity. As a result, multi-cities with varying climatic characteristics will need to conduct more comprehensive studies to investigate the interaction of environmental factors on human health.

The interplay between air pollution and weather conditions was well studied in a Polish study [28]. Specific weather conditions like temperature inversion, lack of wind and precipitation, and high atmospheric pressure trap air pollution in lower parts of the atmosphere, which increases the harmful health effects of air pollution. These harmful health effects include all-cause mortality and pneumonia-related hospitalizations. The researchers found that in Poland, NO_2_ was most likely to increase pneumonia burden. Similarly, since more O_3_ is produced with increased NO_2_, solar radiation intensity, and high temperature, the increase in O_3_ was associated with mortality in both seasons, i.e., summer and winter. PM_2.5_ and wind speed were positively correlated with mortality. The article demonstrated the importance of particular pollutants, their distinct pattern and magnitude, and their interactions with weather conditions. An article by Zhao et al. [44] also noted that NO_2_ and PM_10_ are positively correlated with each other while having inverse and weak associations with O_3_ levels.

Rachel et al., in their review, explained the interplay between environmental factors that may be relevant to the development of food allergies. The relationship between human health, vitamin D synthesis, vegetation, and air pollution is complex and influenced by latitude. Vitamin D synthesis depends on exposure to UVB radiation, which varies with latitude. Greener areas with more vegetation may promote outdoor activities and UVB exposure, but excessive vegetation can block sunlight. Additionally, pollen levels can vary with greenness and air pollution, affecting allergen release and responses. The interplay between air pollution and vegetation is intricate, as vegetation can both mitigate and exacerbate air pollutant exposures, depending on location and structure [148]. In a study conducted in Taiwan, Hong-Ren Yu et al. highlighted the need to thoroughly study and understand how different air pollutants and meteorological conditions combine and affect our surroundings because these interactions have far-reaching consequences that warrant attention and investigation [46].

Argacha et al. [48] highlighted the challenges in accounting for all known and unknown confounding factors in air pollution studies. They emphasized that even though employing various strategies, such as time series studies and case-crossover designs, to address this confounding variables problem, each had its own limitations. While current studies often report the independent effects of individual pollutants or bipollutants, which does not align with the multifactorial nature of air pollution and the potential interactions between pollutants, an optimal method for examining interactions between gaseous and particulate pollutants has yet to be clearly identified. This underscores the need for the scientific community to agree on an optimal screening method that can effectively account for these complex interactions.

Marina et al. found that understanding the specific mechanisms by which environmental and individual factors influence individuals at risk for RA in its preclinical stages is crucial. This knowledge can significantly contribute to early disease diagnosis and, if the identified factor is modifiable, could be instrumental in preventing or delaying the onset of the disease [131]. Wanzhou et al. found that short-term exposures to air pollutants (PM_2.5_, PM_10_, CO, and NO_2_) were linked to increased visits for total eye diseases and conjunctivitis, with stronger effects observed on days with higher temperatures for PM_2.5_, PM_10_, CO, and NO_2_, and on days with lower temperatures for CO and NO_2_. These findings suggest that extreme temperatures exacerbate the harmful effects of air pollution on eye health and highlight the need for targeted protection strategies amid urbanization and climate change [139].

**Figure 12:**
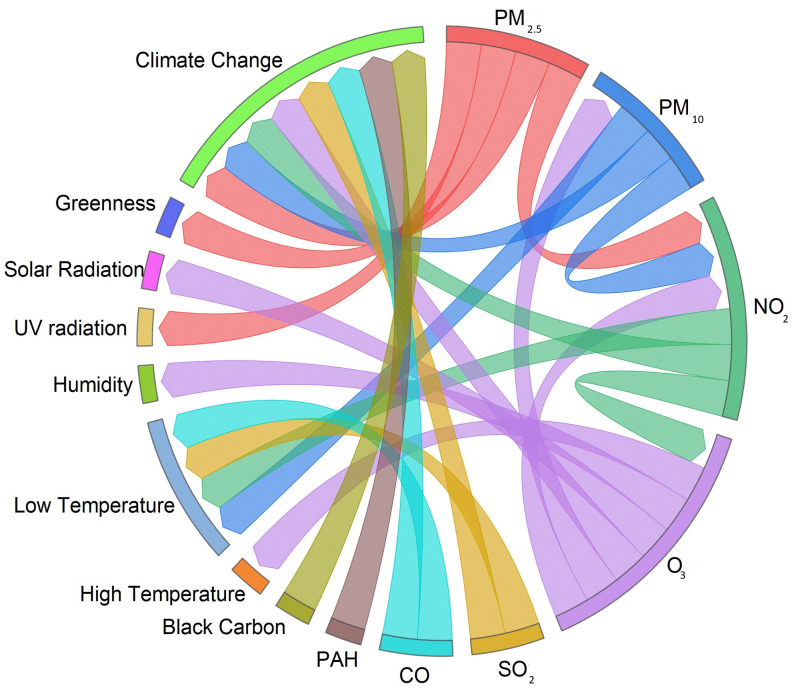
Associations among environmental factors (UV radiation: ultraviolet radiation, PM_2.5_: particulate matter smaller than 2.5 µg/m^3^, PM_10_: particulate matter smaller than 10 µg/m^3^, SO_2_: sulfur dioxide, NO_2_: nitrogen dioxide, O_3_: ozone, CO: carbon monoxide, PAH: polycyclic aromatic hydrocarbon).

Thus, we conclude that the interplay between environmental factors is crucial to all ecological studies, and since these associations are also location-dependent, continuous monitoring and measurement of these factors is necessary. Likewise, future research needs to consider the interrelationships between multiple environmental factors. The chord diagram (Figure 12) depicts the associations among various environmental factors.

### 3.12. Importance of Monitoring Environmental Factors

By reviewing these articles, which covered the majority of general health issues and the most common environmental factors that are associated with these health issues, we recognized the importance of monitoring and surveillance. While surveillance is not a new concept, we found several examples from the literature that suggested that monitoring and surveillance of environmental factors should be carried out to prevent their adverse effects on human health. Jung-Yien Chien et al. [149], while developing a prediction model for acute exacerbation of COPD, utilized sensing devices to gather continuous real-time monitoring data on lifestyle and environmental factors. Their experimental findings supported the notion that environmental data facilitate the patient’s health management and are crucial for developing early warning systems. Since we know that exposure to PM_2.5_ is unavoidable all around the globe, Liu et al. [52] reported that the total exposure to PM_2.5_ increased by 41.2% from 1990 to 2017. These findings and those in [40] highlight the importance of continuously monitoring environmental factors to assess the risk of human health issues.

It is anticipated that extreme weather and climate events will become more frequent and intense over the next decades, potentially having a large impact on people and healthcare systems throughout the world. Before, during, and after disasters, rigorous research may enhance evaluations of population health and health system vulnerabilities and capabilities as well as assist in assessing the efficacy of integrated disaster risk management and adaptation measures. As Bashir et al. [150] suggested, an early warning system using precise remote sensing and GIS technologies with the help of satellite data from the environment is necessary for detecting outbreaks. A system like this will prepare us for future calamities.

Similarly, McKeon et al. [23] suggested that interventions to reduce poor outdoor air quality exposure for those with early-stage lung cancer may improve survival time after lung cancer screening. Their findings supported their hypothesis that air pollution not only causes cancer but also decreases lung cancer survival. Therefore, monitoring approaches or preventive efforts to protect patients from air pollution or other environmental factors involved in the prevalence of the disease after diagnosis are needed. As urbanization and industrialization increase, Xing et al. [33] suggested prioritizing potentially modifiable risk exposure, such as exposure to air pollution.

Even though exposure to green spaces can be beneficial in some cases, like promoting physical activity and reducing psychological stress, it can also cause an increase in wheezing and bronchitis episodes, risk of asthma, and allergic rhinitis. In some cases, though, it is found to reduce poor asthma conditions. This led to Stas et al. [45] suggesting the use of global navigation satellite systems (GNSSs). It was argued that because the health benefits of nature are thought to be dosage-dependent, GNSS tracking provides a more accurate estimate of the actual appropriate dose of nature required by dividing green space types, allergic tree information, and exposure limits by everyone’s special needs. Yang et al. [43] also suggest accurate and dynamic greenness exposure assessments. Dąbrowiecki et al. [28] suggested the data be diverse and encompass individualized exposures, city-specific heterogeneity, and other factors such as demographics and commodities, which impact health collectively.

Since air consists of multiple toxins that are associated with bad respiratory health, Li et al. [151] recommended DEEP (Data-driven ExposurE Profile extraction), a machine learning-based model that uses a high-performing XGBoost algorithm to identify toxins in the air that are associated with asthma. An algorithm like this may prove vital in detecting the co-effects of pollutants that cause certain diseases. Several conventional statistical methods assess environmental factors individually, but toxins in the air and the environment co-exist and affect health outcomes collectively; therefore, DEEP examines air toxin combinations and then determines their association with, in this case, the health issue of childhood asthma.

Similarly, exploring asthma, Yang et al. [37] recommended that due to differences in the natural environment and economic status in different regions, air pollution and disease occurrence also vary between regions. Several studies have shown that different populations respond differently to atmospheric PM. The results confirmed that in children younger than 6 years old, the link between PM concentration and asthma aggravation was more obvious than in older children [37]. Consequently, young children with asthma are more sensitive to the influence of PM, which might be due to the incomplete development of the children’s respiratory and immune systems. This also suggested that more attention needs to be paid to the impact of PM on children, especially young children, when formulating policies on air pollution. This confirms the need for individualized patient exposure measurements.

Jo et al. [39] also proposed that for the proper management of health issues, appropriate interventions that include environmental and personal life management are required. Furthermore, understanding the personal characteristics of individuals who are more susceptible to environmental variables will aid in the creation of appropriate interventions. They also investigated patterns of medical care utilization in individuals with asthma or COPD based on meteorological conditions and air pollution. Although air pollution levels were assessed in regression analysis by place of residence, actual exposure could range significantly from daily average estimates depending on factors such as time spent outside, place of work, and distance between the monitoring station and place of residence. If the distance between the measuring station and your home is large, your actual exposure may differ greatly from the average estimate. Furthermore, the disease’s exacerbation varied. According to these findings, not all patients have a negative impact from environmental elements such as temperature, humidity, and oxygen, on the development of their condition. Therefore, emphasis has also been placed on individualized asthma and COPD care.

Alvaro et al. [40] stressed the importance of monitoring environmental factors due to their association with acute respiratory infections in children in Spain, suggesting that a multivariate study is a necessity. In the literature, they found that some studies presented contradictory evidence regarding the positive relationship between low temperature and high relative humidity and ALRI, but this could be because weather conditions are different in different geographical regions and altitudes, and virus outbreaks also differ seasonally, therefore monitoring environmental and meteorological factors geo-specifically is optimal. In addition, an Iranian study by Razavi et al. [152] assessed the relationship between environmental factors and asthma exacerbations and expressed the importance of a geographical information system because it helps identify the occurrence of a disease and its environmental dependence.

Lastly, Kim et al. [57] surveyed the literature for current knowledge regarding how early-life pollution exposure negatively affects cardiovascular phenotypes and advised early intervention and other strategies that can help prevent this damage. The concepts of lifetime and acute exposure should be well integrated into the health of the general population, and devices to monitor individual and regional exposure should be improved and focused on new exposure dangers. From all this, we conclude that for a better understanding of an environment-affected health issue/disease, continuous monitoring of environmental factors must be geographically location-specific (specifying a region, a city, or a country). Individualized patient exposure should be prioritized. Moreover, modifiable risk factors of diseases should be the primary focus. For example, measures should be taken to reduce air pollution and other controllable risk factors.

On the other hand, adaptive measures should be taken to manage risks, and exposure to non-modifiable environmental factors should be reduced. We saw examples in the literature where improving air quality benefited the health of the general population and pulmonary infections decreased as air quality improved [25,28,48]. Similarly, a decrease in all-cause mortality and cardiovascular mortality was observed with better air quality. Approximately 8000 hospitalizations due to heart attack were prevented by reducing PM_2.5_ concentrations to 3.9 μg/m^3^ [48]. Geospatial analysis can also make it easier to identify the groups most at risk of certain illness outcomes and can provide important information for designing targeted health interventions [153].

The literature survey in this review also included articles that utilized Copernicus satellite data. Copernicus is the European Union’s Earth observation program that provides comprehensive data about our planet and its environment from satellite and ground-based sources. Rodriguez et al. determined the association between PM_2.5_ exposure and COVID-19 mortality [154]. Roberto et al. calculated PM_2.5_ ground-level values in accordance with the location of COVID-19 exposure as stated by the patients so that exposure measurement was specific to an individual’s location [155]. Schneider et al. [156] used Copernicus air quality monitoring service to simulate air pollution concentrations under two emission scenarios: one with COVID-19 restrictions and the other without COVID-19 restrictions. They also applied a spatiotemporal Bayesian non-linear mixed effect model to quantify the changes in pollutant concentrations associated with the stringency indices of individual policy measures. They aimed to observe the effects of the lockdown on air pollution levels and CAMS provided the solution.

Beloconi et al. [157] also used Copernicus satellite data to measure annual averages of NO_2_ concentrations in μg/m^3^ and used a Bayesian geostatistical model for the estimation of air pollution exposure at high spatial resolution. De et al. [158] demonstrated that by using earth observation data, we can develop robust models for forecasting and modeling temporal (daily) variations in several pollutants, such as NO_2_. These predictions can help with medical research. Similarly, Jacobson et al. [159] extracted daily mean temperature and relative humidity data for their study to find an association between daily average temperature and respiratory mortality in elderly Brazilians.

In [160], while measuring mean NO_2_ concentration exposure in participants who shared their location data, Gignac et al. used air quality estimates from the Copernicus satellite data when the participant moved outside Barcelona or away from ground NO_2_-exposure measurement stations. These studies underscore the need for enhanced centralized monitoring and surveillance systems that are accessible to everyone. Further details related to the Copernicus health project and literature are given in Supplementary File S1 of this review.

## 4. Discussion

The main idea behind this review was to explore the effects of major environmental factors on general human health. Even though there is overwhelming evidence of the human environment affecting human health in the literature, the review papers written in this domain are in highly focused areas of research discussing specific environmental factors and specific human illnesses [48]. A research gap was identified while assessing the literature: there is a lack of a comprehensive review that would provide a broad perspective of the effects of the environment on general human health. Since we are continuously exposed to them, environmental factors greatly determine our well-being and can in no way be ignored when human health is under consideration. Broadly, everything we are exposed to affects our bodies and it is practically impossible to consider the effect of every individual factor on human health. That is why a need was felt for a broader categorization of both the environmental factors and the diseases that they cause.

The keyword search disclosed that the majority of the literature is focused on exploring associations between a specific environmental factor and a specific disease in a specific geographical region. The article pool we obtained was highly diverse and contained articles about associations between different environmental factors and diseases.

An important phenomenon observed while reviewing these articles was the mutual interactions or interplay of environmental factors with each other. It was learned from the literature that the interactions among environmental factors are very crucial and may cause several physiological complications in humans. For instance, the interplay and interaction between air pollutants and weather conditions act as a secondary exposure or influence and increase risk intensity. Apart from the obvious interactions like solar radiation and ozone, there are certain non-obvious interactions at play, such as humidity, ozone, etc. To understand the health and environmental effects of these exposures accurately, researchers and policymakers need to consider the fact that individuals are exposed to multiple environmental factors simultaneously. This is what is referred to as the “multipollutant exposure phenomenon”. In essence, it emphasizes the importance of studying and regulating environmental factors by considering the combined impact of various pollutants rather than looking at each pollutant separately [38,46].

As discussed above, we commenced the review process by categorizing health issues into nine sections, which helped us generalize human health. Evidence in the literature showed that several environmental factors like air pollution, weather conditions, solar radiation, climate change, and greenness are associated with these nine categories of human health issues. We found several environmental factors affecting respiratory health. External environmental factors like air pollution, temperature, and greenness were found to affect several respiratory diseases such as COPD, AE-IPF, ALRI, asthma, hay fever, pneumonia, and lung cancer. Similarly, external environmental factors such as air pollution and temperature were found to have associations with cardiovascular mortality and morbidity. Several serious and long-term cardiovascular health issues like hypertension, heart attack, stroke, heart failure, cardiac arrest, arrhythmia, atrial fibrillation, and atherosclerosis were found to be affected by increased levels of air pollution and low temperatures. Furthermore, the reviewed literature demonstrated that external environmental factors like air pollution are an important risk factor for metabolic and gastrointestinal health issues. In addition, we found that air pollution, climate change, and weather conditions greatly affect mental health. They were found to increase certain mental problems like anxiety, depression, nervousness, suicide, low mood, schizophrenia, bipolar affective disorder, psychosis, PTSD, cognitive dysfunction, neuro-inflammation, low mood, stress, cognitive impairment, and adverse neurobehavioral outcomes. On the other hand, greenness was associated with improved mental health and reduced stress.

Similarly, we also found air pollution causing serious health issues like IBS, peptic ulcers, noninfectious gastroenteritis, liver disease, diabetes, renal failure, renal colic, appendicitis, gastritis, duodenitis, esophageal disorders, and intestinal infections. We discovered that certain environmental factors like air pollution, specifically PM_2.5_ and PM_10_, NO_2_, and O_3_, weather conditions such as humidity, wind speed, and average temperature, CH_4_, and greenness are correlated with several infectious and skin diseases such as angioedema, COVID-19, chicken pox, dengue, tuberculosis, measles, and atopic eczema. We learned that air pollution, weather conditions, solar radiation, and greenness were found to be associated with certain cancers like skin, lung, oral, esophagus, stomach, colon, rectal, liver, gall bladder, larynx, bone, breast, cervix, prostate, blood, gastrointestinal, and brain cancers.

We also found several articles relating to the risk factors of climate change and how it affects human health [141,142,143,144,145,146]. The main highlight of this observation was that climate change increases the risks and intensities of not only respiratory, cardiovascular, and mental health issues but also the chances of infectious diseases. Moreover, climate change greatly increases the level of air pollution in the atmosphere and is also the reason for the increase in extreme temperature fluctuations that have adverse effects on every health category that we have defined above.

Several factors contribute to the development and exacerbation of a disease; these factors vary depending on geographical location, environmental factors, and individual lifestyle. Identifying these diseases aids in their prevention. As a result, the role of various environmental factors in the occurrence and exacerbation of a disease can be measured by collecting appropriate information about an individual’s living environment. The location or place where something is happening or being measured can indirectly represent or stand in for other factors or variables that were not directly measured or considered in the study [41].

Therefore, we found that geo-localized continuous monitoring of environmental factors is necessary for a better understanding of human health. We also learned that satellite-based services like Copernicus utilized satellite and non-satellite information and were able to predict and help in the management of health issues. These projects also depicted how the monitoring approaches help prevent certain diseases by using alarm systems. The literature showed that these systems greatly reduced emergency hospitalizations and helped the medical care system perform better in case of disasters or infectious outbreaks [157,158,161,162].

While conducting the review, we discovered that most of the studies were about air pollution. Evidence in the literature says that air pollution has the greatest impact of any environmental factor on human health. It is a potential environmental hazard and a major risk factor for a variety of respiratory and cardiovascular diseases. Components of air pollution also interact with one another, acting as secondary pollutants. Knowledge of air pollution levels, weather conditions, and geographical location is essential for measuring associations between environmental factors and general human health. These associations must be location-based in order to have a numerical value. It was observed that the geographical location was fixed for almost every study that found an association between an environmental factor and a disease. Because air pollution and weather conditions differ by geographical location, it is interesting to note that these environmental factors interact with each other differently in different locations. This suggests that these inter-relationships may be stronger in some areas than in others.

We also discovered that sensitivity to environmental factors tends to vary among diseases. Recognizing the modifiable and non-modifiable risk factors of a disease can help reduce the disease burden. The first step is to increase awareness, followed by avoidance of non-modifiable risk factors and reduction of exposure to modifiable risk factors. We also saw, among these studies, that the majority concerned developed countries or cities. Some of these modern cities and countries where these studies were carried out include developed regions like Spain, France, and Switzerland in Europe; China and Japan in Asia, and Ohio and California in the USA. Therefore, there is a pressing need to carry out extensive environmental and health-related studies in LMICs [53]. One of the reasons for the lower number of studies in LMICs is a lack of advanced geo-sensors. For this reason, it was challenging to collect environmental data in underdeveloped regions. Even though, in this case, Copernicus was a great help in providing environmental information through satellites, more studies are required in underdeveloped regions because these regions are also a bigger target for the climate crisis. Studies that correlate environmental factors with human health are needed and beneficial for a healthier future for LMICs.

**Figure 13:**
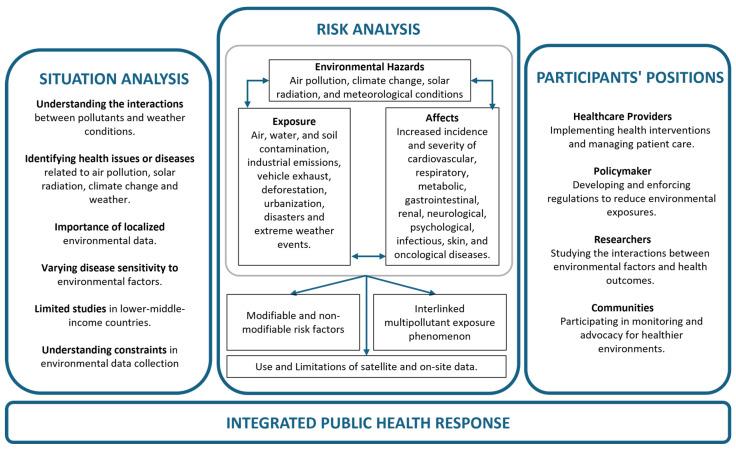
Integrated public health risk assessment and response framework.

Adopting an integrated approach to public health risk assessment, as illustrated in Figure 13, can help systematically address the complex interplay of environmental factors. The adapted diagram, inspired by Mahoney et al.’s Figure 1 [163], demonstrates the comprehensive approach needed to monitor and manage the health impacts of environmental factors.

Within the limits of our search, we were able to identify DEEP [151], along with other statistical models such as the spatio-temporal Bayesian non-linear mixed effect model, a Bayesian geostatistical model, and other machine learning models used to analyze the relationship between environmental factors and health. Moreover, Linling et al. discussed practical statistical methods commonly employed in environmental epidemiology to assess the health effects of exposure to multi-pollutant mixtures. These methods include Bayesian kernel machine regression (BKMR), weighted quantile sum (WQS) regressions, shrinkage techniques (such as least absolute shrinkage and selection operator, the elastic network model, the adaptive elastic-net model, and principal component analysis), and environment-wide association studies (EWASs) [164]. It is worth noting that the choice of the most suitable model can vary depending on the specific study’s data and objectives, as well as the unique characteristics of each statistical approach. Encouragingly, ongoing advancements in the field are driving the development of more sophisticated methods to elucidate the intricate relationships between environmental determinants and health outcomes.

## 5. Limitations

This review, due to its comprehensive approach, does come with some limitations. In our pursuit to uncover a broad range of associations between human health issues and the environment, we made the deliberate choice not to include micro-level disease-specific information or delve deeply into individual environmental factors. For example, we did not cover pesticide-related pollution and its health impacts, even though exposure to volatile organic compounds (VOCs) from pesticides is known to affect health [165]. Due to the need to maintain a manageable scope, we could not cover every conceivable health issue, choosing instead to focus on those that appear most prevalent in the majority of the related literature.

Our analysis primarily concentrated on exploring positive links between environmental factors and health issues, but negative links or non-associations were not considered. While this approach provided valuable insights into potential associations, it is essential to recognize that the omission of negative links or non-associations may introduce a bias in our findings. In future research, a more comprehensive assessment, including both positive and negative associations, would offer a more complete picture of the complex interplay between environmental factors and health issues.

While the studies utilized satellite data to investigate environmental factors and their impact on human health, utilizing satellite data has limitations in terms of spatial resolution. This constraint may limit the ability to capture fine-scale variations and nuances in environmental factors.

## 6. Conclusions

This review highlights the significant impact of various environmental factors, such as air pollution, weather conditions, and climate change, on human health. These factors affect multiple health outcomes, including cardiovascular, respiratory, mental, metabolic and gastrointestinal, renal and urogenital, neurological, and psychological health, as well as the risk of infectious and skin diseases and major types of cancers. The interplay between these factors further amplifies their effects. We recommend that individualized treatment and management for targeted prevention of disease based on continuous localized monitoring using GIS and satellite data (e.g., Copernicus) is crucial for the management of diseases caused by environmental factors.

## Data Availability

Detailed categorization of the manuscripts reviewed will be shared upon reasonable request after the publication of the original paper.

## Supplementary Materials

The following supporting information can be downloaded at: https://www.mdpi.com/article/10.3390/healthcare12212123/s1, Table S1: Summary of the reviewed manuscripts focused on the effects of major environmental factors on general human health: the diseases discussed under the influence of the environment, the manuscripts containing satellite or Copernicus data, and the environmental factors examined in the context of human health; Supplementary File S1: Health-related projects that utilize Copernicus’s environmental data [98,155,156,157,158,159,161,162,166,167,168,169,170,171,172,173,174,175,176,177,178].

## References

[1] Wang X., Wu F., Zhao X., Zhang X., Wang J., Niu L., Liang W., Leung K.M.Y., Giesy J.P. (2022). Enlightenment from the COVID-19 pandemic: The roles of environmental factors in future public health emergency response. Engineering.

[2] Riggs D.W., Yeager R.A., Bhatnagar A. (2018). Defining the human envirome: An omics approach for assessing the environmental risk of cardiovascular disease. Circ. Res..

[3] Alabi O.A., Ologbonjaye K.I., Awosolu O., Alalade O.E. (2019). Public and environmental health effects of plastic wastes disposal: A review. J. Toxicol. Risk Assess..

[4] de Prado Bert P., Mercader E.M.H., Pujol J., Sunyer J., Mortamais M. (2018). The effects of air pollution on the brain: A review of studies interfacing environmental epidemiology and neuroimaging. Curr. Environ. Health Rep..

[5] Menon J., Struijs F., Whaley P. (2022). The methodological rigour of systematic reviews in environmental health. Crit. Rev. Toxicol..

[6] Cleary E.G., Patton A.P., Wu H.-C., Xie A., Stubblefield J., Mass W., Grinstein G., Koch-Weser S., Brugge D., Wong C. (2017). Making air pollution visible: A tool for promoting environmental health literacy. JMIR Public Health Surveill..

[7] Schulz A., Northridge M.E. (2004). Social Determinants of Health: Implications for Environmental Health Promotion. Health Educ. Behav..

[8] Santos S., Maitre L., Warembourg C., Agier L., Richiardi L., Basagaña X., Vrijheid M. (2020). Applying the exposome concept in birth cohort research: A review of statistical approaches. Eur. J. Epidemiol..

[9] Vos T., Barber R.M., Bell B., Bertozzi-Villa A., Biryukov S., Bolliger I., Charlson F., Davis A., Degenhardt L., Dicker D. (2015). Global, regional, and national incidence, prevalence, and years lived with disability for 301 acute and chronic diseases and injuries in 188 countries, 1990–2013: A systematic analysis for the Global Burden of Disease Study 2013. Lancet.

[10] Ma Y., Zhang Y., Cheng B., Feng F., Jiao H., Zhao X., Ma B., Yu Z. (2020). A review of the impact of outdoor and indoor environmental factors on human health in China. Environ. Sci. Pollut. Res..

[11] Pryor J.T., Cowley L.O., Simonds S.E. (2022). The Physiological Effects of Air Pollution: Particulate Matter, Physiology and Disease. Front. Public Health.

[12] Fuller R., Landrigan P.J., Balakrishnan K., Bathan G., Bose-O’Reilly S., Brauer M., Caravanos J., Chiles T., Cohen A., Corra L. (2022). Pollution and health: A progress update. Lancet Planet. Health.

[13] Goldman L., Schafer A.I. (2019). Goldman-Cecil Medicine E-Book.

[14] Williamson P.O., Minter C.I. (2019). Exploring PubMed as a reliable resource for scholarly communications services. J. Med. Libr. Assoc. JMLA.

[15] Shin S., Bai L., Burnett R.T., Kwong J.C., Hystad P., van Donkelaar A., Lavigne E., Weichenthal S., Copes R., Martin R.V. (2021). Air pollution as a risk factor for incident chronic obstructive pulmonary disease and asthma. A 15-year population-based cohort study. Am. J. Respir. Crit. Care Med..

[16] Crouse D.L., Pinault L., Balram A., Brauer M., Burnett R.T., Martin R.V., van Donkelaar A., Villeneuve P.J., Weichenthal S. (2019). Complex relationships between greenness, air pollution, and mortality in a population-based Canadian cohort. Environ. Int..

[17] Tsai H.-J., Li C.-Y., Pan W.-C., Yao T.-C., Su H.-J., Wu C.-D., Chern Y.-R., Spengler J.D. (2021). The effect of surrounding greenness on type 2 diabetes mellitus: A nationwide population-based cohort in Taiwan. Int. J. Environ. Res. Public Health.

[18] Lin S.-Y., Ju S.-W., Hsu W.-H., Lin C.-C., Ting I.-W., Kao C.-H. (2020). Air pollutants and subsequent risk of chronic kidney disease and end-stage renal disease: A population-based cohort study. Environ. Pollut..

[19] Chen S.-F., Chien Y.-H., Chen P.-C. (2023). The association between long-term ambient fine particulate exposure and the mortality among adult patients initiating dialysis: A retrospective population-based cohort study in Taiwan. Environ. Pollut..

[20] Culqui Lévano D.R., Díaz J., Blanco A., Lopez J.A., Navas M.A., Sánchez-Martínez G., Luna M.Y., Hervella B., Belda F., Linares C. (2022). Mortality due to COVID-19 in Spain and its association with environmental factors and determinants of health. Environ. Sci. Eur..

[21] Santos U.D.P., Arbex M.A., Braga A.L.F., Mizutani R.F., Cancado J.E.D., Terra-Filho M., Chatkin J.M. (2021). Environmental air pollution: Respiratory effects. J. Bras. Pneumol..

[22] World Health Organization (WHO) (2013). Outdoor air pollution a leading environmental cause of cancer deaths. IARC Sci. Publ..

[23] McKeon T.P., Vachani A., Penning T.M., Hwang W.T. (2022). Air pollution and lung cancer survival in Pennsylvania. Lung Cancer.

[24] So R., Andersen Z.J., Chen J., Stafoggia M., de Hoogh K., Katsouyanni K., Vienneau D., Rodopoulou S., Samoli E., Lim Y.-H. (2022). Long-term exposure to air pollution and mortality in a Danish nationwide administrative cohort study: Beyond mortality from cardiopulmonary disease and lung cancer. Environ. Int..

[25] Aix M.L., Petit P., Bicout D.J. (2022). Air pollution and health impacts during the COVID 19 lockdowns in Grenoble, France. Environ. Pollut..

[26] Lefler J.S., Higbee J.D., Burnett R.T., Ezzati M., Coleman N.C., Mann D.D., Marshall J.D., Bechle M., Wang Y., Robinson A.L. (2019). Air pollution and mortality in a large, representative US cohort: Multiple-pollutant analyses, and spatial and temporal decompositions. Environ. Health.

[27] Zhao H., Chen S., Yang F., Wu H., Ba Y., Cui L., Chen R., Zhu J. (2022). Alternation of nasopharyngeal microbiota in healthy youth is associated with environmental factors: Implication for respiratory diseases. Int. J. Environ. Health Res..

[28] Dąbrowiecki P., Badyda A., Chciałowski A., Czechowski P.O., Wrotek A. (2022). Influence of Selected Air Pollutants on Mortality and Pneumonia Burden in Three Polish Cities over the Years 2011–2018. J. Clin. Med..

[29] Yang M., Guo Y.-M., Bloom M.S., Dharmagee S.C., Morawska L., Heinrich J., Jalaludin B., Markevychd I., Knibbsf L.D., Lin S. (2020). Is PM1 similar to PM2.5? A new insight into the association of PM1 and PM2.5 with children’s lung function. Environ. Int..

[30] Han W., Su Y., Liu B., Zhu W., Yu X., Sun X., Qi X., Lin X., Rizvi S.A.A., Song W.-J. (2022). Association between hospitalizations for asthma exacerbation and weather conditions in Qingdao: An ecological study. Ann. Transl. Med..

[31] Khatri S.B., Newman C., Hammel J.P., Dey T., Van Laere J.J., Ross K.A., Rose J.A., Anderson T., Mukerjee S., Smith L. (2021). Associations of air pollution and pediatric asthma in Cleveland, Ohio. Sci. World J..

[32] Wang C., Wei C.C., Wan L., Lin C.L., Tsai J.D. (2021). Association of exposure to hydrocarbon air pollution with the incidence of atopic dermatitis in children. Ital. J. Pediatr..

[33] Xing Y., Wong G.W.K. (2022). Environmental Influences and Allergic Diseases in the Asia-Pacific Region: What Will Happen in Next 30 Years?. Allergy Asthma Immunol. Res..

[34] Norbäck D., Lu C., Zhang Y., Li B., Zhao Z., Huang C., Zhang X., Qian H., Sun Y., Wang J. (2019). Sources of indoor particulate matter (PM) and outdoor air pollution in China in relation to asthma, wheeze, rhinitis and eczema among pre-school children: Synergistic effects between antibiotics use and PM10 and second hand smoke. Environ. Int..

[35] Tahara M., Fujino Y., Yamasaki K., Oda K., Kido T., Sakamoto N., Kawanami T., Kataoka K., Egashira R., Hashisako M. (2021). Exposure to PM2.5 is a risk factor for acute exacerbation of surgically diagnosed idiopathic pulmonary fibrosis: A case control study. Respir. Res..

[36] Orellano P., Quaranta N., Reynoso J., Balbi B., Vasquez J. (2018). Association of outdoor air pollution with the prevalence of asthma in children of Latin America and the Caribbean: A systematic review and meta-analysis. J. Asthma.

[37] Yang X., Zhang Y., Zhan X., Xu X., Li S., Xu X., Ying S., Chen Z. (2021). Particulate matter exposure is highly correlated to pediatric asthma exacerbation. Aging.

[38] Yamazaki S., Shima M., Yoda Y., Oka K., Kurosaka F., Shimizu S., Takahashi H., Nakatani Y., Nishikawa J., Fujiwara K. (2015). Exposure to air pollution and meteorological factors associated with children’s primary care visits at night due to asthma attack: Case-crossover design for 3-year pooled patients. BMJ Open.

[39] Jo E.-J., Choi M.-H., Kim C.-H., Won K.-M., Kim Y.-K., Jeong J.-H., An H.Y., Hwang M.-K., Park H.-K. (2021). Patterns of medical care utilization according to environmental factors in asthma and chronic obstructive pulmonary disease patients. Korean J. Intern. Med..

[40] Álvaro-Meca A., del Carmen Goez M., Resino R., Matías V., Sepúlveda-Crespo D., Martínez I., Resino S. (2022). Environmental factors linked to hospital admissions in young children due to acute viral lower respiratory infections: A bidirectional case crossover study. Environ. Res..

[41] Mentz G., Robins T.G., Batterman S., Naidoo R.N. (2019). Effect modifiers of lung function and daily air pollutant variability in a panel of schoolchildren. Thorax.

[42] Wu R., Guo Q., Fan J., Guo C., Wang G., Wu W., Xu J. (2022). Association between air pollution and outpatient visits for allergic rhinitis: Effect modification by ambient temperature and relative humidity. Sci. Total Environ..

[43] Yang B.-Y., Zhao T., Hu L.-X., Browning M.H., Heinrich J., Dharmage S.C., Jalaludin B., Knibbs L.D., Liu X.-X., Luo Y.-N. (2021). Greenspace and human health: An umbrella review. Innovation.

[44] Zhao T., Markevych I., Standl M., Lyu Z., Schikowski T., Berdel D., Koletzko S., von Berg A., Heinrich J. (2022). Ambient ozone exposure and bone turnover markers in children: Results from the GINIplus and LISA birth cohorts. Environ. Res..

[45] Stas M., Aerts R., Hendrickx M., Delcloo A., Dendoncker N., Dujardin S., Linard C., Nawrot T., Van Nieuwenhuyse A., Aerts J.-M. (2021). Exposure to green space and pollen allergy symptom severity: A case-crossover study in Belgium. Sci. Total Environ..

[46] Yu H.-R., Lin C.-H.R., Tsai J.-H., Hsieh Y.-T., Tsai T.-A., Tsai C.-K., Lee Y.-C., Liu T.-Y., Tsai C.-M., Chen C.-C. (2020). A multifactorial evaluation of the effects of air pollution and meteorological factors on asthma exacerbation. Int. J. Environ. Res. Public Health.

[47] Kim H., Kim H., Lee J.T. (2018). Assessing the cold temperature effect on hospital visit by allergic rhinitis in Seoul, Korea. Sci. Total Environ..

[48] Argacha J.F., Bourdrel T., Van De Borne P. (2018). Ecology of the cardiovascular system: A focus on air-related environmental factors. Trends Cardiovasc. Med..

[49] de Bont J., Jaganathan S., Dahlquist M., Persson Å., Stafoggia M., Ljungman P. (2022). Ambient air pollution and cardiovascular diseases: An umbrella review of systematic reviews and meta-analyses. J. Intern. Med..

[50] Niu Z., Liu F., Yu H., Wu S., Xiang H. (2021). Association between exposure to ambient air pollution and hospital admission, incidence, and mortality of stroke: An updated systematic review and meta-analysis of more than 23 million participants. Environ. Health Prev. Med..

[51] Münzel T., Daiber A. (2018). Environmental stressors and their impact on health and disease with focus on oxidative stress. Antioxid. Redox Signal..

[52] Liu Y., Zhao D., Peng W., Xue P., Jiang X., Chen S., Gao H., Wang X., Feng S. (2021). Atmospheric PM2.5 blocking up autophagic flux in HUVECs via inhibiting Sntaxin-17 and LAMP2. Ecotoxicol. Environ. Saf..

[53] Pena M.S.B., Rollins A. (2017). Environmental exposures and cardiovascular disease: A challenge for health and development in low-and middle-income countries. Cardiol. Clin..

[54] Daiber A., Lelieveld J., Steven S., Oelze M., Kröller-Schön S., Sørensen M., Münzel T. (2019). The ‘exposome’ concept–how environmental risk factors influence cardiovascular health. Acta Biochim. Pol..

[55] Jalali S., Karbakhsh M., Momeni M., Taheri M., Amini S., Mansourian M., Sarrafzadegan N. (2021). Long-term exposure to PM2.5 and cardiovascular disease incidence and mortality in an Eastern Mediterranean country: Findings based on a 15-year cohort study. Environ. Health.

[56] Hayes R.B., Lim C., Zhang Y., Cromar K., Shao Y., Reynolds H.R., Silverman D.T., Jones R.R., Park Y., Jerrett M. (2020). PM2.5 air pollution and cause-specific cardiovascular disease mortality. Int. J. Epidemiol..

[57] Kim J.B., Prunicki M., Haddad F., Dant C., Sampath V., Patel R., Smith E., Akdis C., Balmes J., Snyder M.P. (2020). Cumulative lifetime burden of cardiovascular disease from early exposure to air pollution. J. Am. Heart Assoc..

[58] Du W., Wang J., Zhang S., Fu N., Yang F., Wang G., Wang Z., Mao K., Shen G., Qi M. (2021). Impacts of Chinese spring festival on household PM2.5 pollution and blood pressure of rural residents. Indoor Air.

[59] Byrd J.B., Morishita M., Bard R.L., Das R., Wang L., Sun Z., Spino C., Harkema J., Dvonch J.T., Rajagopalan S. (2016). Acute increase in blood pressure during inhalation of coarse particulate matter air pollution from an urban location. J. Am. Soc. Hypertens..

[60] Yang B.-Y., Qian Z., Howard S.W., Vaughn M.G., Fan S.-J., Liu K.-K., Dong G.-H. (2018). Global association between ambient air pollution and blood pressure: A systematic review and metaanalysis. Environ. Pollut..

[61] Aronson D. (2021). Environmental factors, winter respiratory infections and the seasonal variation in heart failure admissions. Sci. Rep..

[62] Wang X., Li G., Liu L., Westerdahl D., Jin X., Pan X. (2015). Effects of extreme temperatures on cause-specific cardiovascular mortality in China. Int. J. Environ. Res. Public Health.

[63] Yeager R.A., Smith T.R., Bhatnagar A. (2020). Green environments and cardiovascular health. Trends Cardiovasc. Med..

[64] Guo M., Zhou M., Li B., Du C., Yao R., Wang L., Yang X., Yu W. (2022). Reducing indoor relative humidity can improve the circulation and cardiorespiratory health of older people in a cold environment: A field trial conducted in Chongqing, China. Sci. Total Environ..

[65] Loo E.X.L., Wang D.Y., Siah K.T.H. (2020). Association between irritable bowel syndrome and allergic diseases: To make a case for aeroallergen. Int. Arch. Allergy Immunol..

[66] Gu J., Shi Y., Zhu Y., Chen N., Wang H., Zhang Z., Chen T. (2020). Ambient air pollution and cause-specific risk of hospital admission in China: A nationwide time-series study. PLoS Med..

[67] Wu M., Lu J., Yang Z., Wei F., Shen P., Yu Z., Tang M., Jin M., Lin H., Chen K. (2021). Ambient air pollution and hospital visits for peptic ulcer disease in China: A three-year analysis. Environ. Res..

[68] Seo H.S., Hong J., Jung J. (2020). Relationship of meteorological factors and air pollutants with medical care utilization for gastroesophageal reflux disease in urban area. World J. Gastroenterol..

[69] Ghaffari H.R., Yunesian M., Nabizadeh R., Nasseri S., Sadjadi A., Pourfarzi F., Poustchi H., Eshraghian A. (2019). Environmental etiology of gastric cancer in Iran: A systematic review focusing on drinking water, soil, food, radiation, and geographical conditions. Environ. Sci. Pollut. Res..

[70] Joyce E.Y., Mallapaty A., Miller R.L. (2018). It’s not just the food you eat: Environmental factors in the development of food allergies. Environ. Res..

[71] Davis R.E., Driskill E.K., Novicoff W.M. (2022). The association between weather and emergency department visitation for diabetes in Roanoke, Virginia. Int. J. Biometeorol..

[72] Yang B.-Y., Fan S., Thiering E., Seissler J., Nowak D., Dong G.-H., Heinrich J. (2020). Ambient air pollution and diabetes: A systematic review and meta-analysis. Environ. Res..

[73] Wei Y., Wang Y., Di Q., Choirat C., Wang Y., Koutrakis P., Zanobetti A., Dominici F., Schwartz J.D. (2019). Short term exposure to fine particulate matter and hospital admission risks and costs in the Medicare population: Time stratified, case crossover study. BMJ.

[74] Yang B.-Y., Markevych I., Heinrich J., Bowatte G., Bloom M.S., Guo Y., Dharmage S.C., Jalaludin B., Knibbs L.D., Morawska L. (2019). Associations of greenness with diabetes mellitus and glucose-homeostasis markers: The 33 Communities Chinese Health Study. Int. J. Hyg. Environ. Health.

[75] O’Donovan G., Chudasama Y., Grocock S., Leigh R., Dalton A.M., Gray L.J., Yates T., Edwardson C., Hill S., Henson J. (2017). The association between air pollution and type 2 diabetes in a large cross-sectional study in Leicester: The CHAMPIONS Study. Environ. Int..

[76] Yang H.Y., Lee J.K.W. (2021). The impact of temperature on the risk of COVID-19: A multinational study. Int. J. Environ. Res. Public Health.

[77] Chen Y., Cao F., Xiao J.P., Fang X.Y., Wang X.R., Ding L.H., Wang D.G., Pan H.F. (2021). Emerging role of air pollution in chronic kidney disease. Environ. Sci. Pollut. Res..

[78] Hwang S.Y., Jeong S., Choi S., Kim D.H., Kim S.R., Lee G., Son J.S., Park S.M. (2021). Association of air pollutants with incident chronic kidney disease in a nationally representative cohort of Korean adults. Int. J. Environ. Res. Public Health.

[79] Liu H., Shao X., Jiang X., Liu X., Bai P., Lin Y., Chen J., Hou F., Cui Z., Zhang Y. (2022). Joint exposure to outdoor ambient air pollutants and incident chronic kidney disease: A prospective cohort study with 90,032 older adults. Front. Public Health.

[80] Copur S., Ucku D., Kanbay M. (2022). Increase in the global burden of chronic kidney disease: Might it be attributable to air pollution?. Clin. Kidney J..

[81] Wu C.D., Chern Y.R., Pan W.C., Lung S.C.C., Yao T.C., Tsai H.J., Spengler J.D. (2020). Effects of surrounding environment on incidence of end stage renal disease. Sci. Total Environ..

[82] Kim J., Kim H. (2017). Demographic and environmental factors associated with mental health: A cross-sectional study. Int. J. Environ. Res. Public Health.

[83] Chen K., Zhang T., Liu F., Zhang Y., Song Y. (2021). How does urban green space impact residents’ mental health: A literature review of mediators. Int. J. Environ. Res. Public Health.

[84] Gu H., Yan W., Elahi E., Cao Y. (2020). Air pollution risks human mental health: An implication of two-stages least squares estimation of interaction effects. Environ. Sci. Pollut. Res..

[85] Chen B., Ma W., Pan Y., Guo W., Chen Y. (2021). PM2.5 exposure and anxiety in China: Evidence from the prefectures. BMC Public Health.

[86] He G., Chen Y., Wang S., Dong Y., Ju G., Chen B. (2020). The association between PM2.5 and depression in China. Dose-Response.

[87] Chen Y., He G., Chen B., Wang S., Ju G., Ge T. (2020). The association between PM2.5 exposure and suicidal ideation: A prefectural panel study. BMC Public Health.

[88] Turner A.L., Brokamp C., Wolfe C., Reponen T., Brunst K.J., Ryan P.H. (2022). Mental and Physical Stress Responses to Personal Ultrafine Particle Exposure in Adolescents. Int. J. Environ. Res. Public Health.

[89] Xue T., Zhu T., Zheng Y., Zhang Q. (2019). Declines in mental health associated with air pollution and temperature variability in China. Nat. Commun..

[90] Cruz J., Li G., Aragon M.J., Coventry P.A., Jacobs R., Prady S.L., White P.C.L. (2022). Association of environmental and socioeconomic indicators with serious mental illness diagnoses identified from general practitioner practice data in England: A spatial Bayesian modelling study. PLoS Med..

[91] Gao W., Tu R., Li H., Fang Y., Que Q. (2020). In the Subtropical Monsoon Climate High-Density City, What Features of the Neighborhood Environment Matter Most for Public Health?. Int. J. Environ. Res. Public Health.

[92] Aguglia A., Serafini G., Escelsior A., Amore M., Maina G. (2020). What is the role of meteorological variables on involuntary admission in psychiatric ward? An Italian cross-sectional study. Environ. Res..

[93] Cianconi P., Betrò S., Janiri L. (2020). The impact of climate change on mental health: A systematic descriptive review. Front. Psychiatry.

[94] Palinkas L.A., Wong M. (2020). Global climate change and mental health. Curr. Opin. Psychol..

[95] Luong T.T., Handley T., Austin E.K., Kiem A.S., Rich J.L., Kelly B. (2021). New Insights Into the Relationship Between Drought and Mental Health Emerging From the Australian Rural Mental Health Study. Front. Psychiatry.

[96] Batterham P.J., Brown K., Trias A., Poyser C., Kazan D., Calear A.L. (2022). Systematic review of quantitative studies assessing the relationship between environment and mental health in rural areas. Aust. J. Rural Health.

[97] Mora C., McKenzie T., Gaw I.M., Dean J.M., von Hammerstein H., Knudson T.A., Setter R.O., Smith C.Z., Webster K.M., Patz J.A. (2022). Over half of known human pathogenic diseases can be aggravated by climate change. Nat. Clim. Chang..

[98] Ma Y., Zhou J., Yang S., Zhao Y., Zheng X. (2017). Assessment for the impact of dust events on measles incidence in western China. Atmos. Environ..

[99] Chen B., Sumi A., Wang L., Zhou W., Kobayashi N. (2017). Role of meteorological conditions in reported chickenpox cases in Wuhan and Hong Kong, China. BMC Infect. Dis..

[100] Li T., Yang Z., Wang M. (2013). Temperature, relative humidity and sunshine may be the effective predictors for occurrence of malaria in Guangzhou, southern China, 2006–2012. Parasites Vectors.

[101] Xiang J., Hansen A., Liu Q., Liu X., Tong M.X., Sun Y., Cameron S., Hanson-Easey S., Han G.-S., Williams C. (2017). Association between dengue fever incidence and meteorological factors in Guangzhou, China, 2005–2014. Environ. Res..

[102] Fan J., Lin H., Wang C., Bai L., Yang S., Chu C., Yang W., Liu Q. (2014). Identifying the high-risk areas and associated meteorological factors of dengue transmission in Guangdong Province, China from 2005 to 2011. Epidemiol. Infect..

[103] Jaakkola K., Saukkoriipi A., Jokelainen J., Juvonen R., Kauppila J., Vainio O., Ziegler T.T., Rönkkö E., Jaakkola J.J.K., Ikäheimo T.M. (2014). Decline in temperature and humidity increases the occurrence of influenza in cold climate. Environ. Health.

[104] Zoran M.A., Savastru R.S., Savastru D.M., Tautan M.N. (2020). Assessing the relationship between surface levels of PM2.5 and PM10 particulate matter impact on COVID-19 in Milan, Italy. Sci. Total Environ..

[105] Fang F., Mu L., Zhu Y., Rao J., Heymann J., Zhang Z.F. (2021). Long-term exposure to PM2.5, facemask mandates, stay home orders and COVID-19 incidence in the United States. Int. J. Environ. Res. Public Health.

[106] Huang K., Hu C.-Y., Yang X.-Y., Zhang Y., Wang X.-Q., Zhang K.-D., Li Y.-Q., Wang J., Yu W.-J., Cheng X. (2022). Contributions of ambient temperature and relative humidity to the risk of tuberculosis admissions: A multicity study in Central China. Sci. Total Environ..

[107] Sharma A., Verma H.K., Joshi S., Panwar M.S., Mandal C.C. (2015). A link between cold environment and cancer. Tumor Biol..

[108] Lucas R., Norval M., Neale R.E., Young A.R., de Gruijl F.R., Takizawa Y., van der Leun J.C. (2015). The consequences for human health of stratospheric ozone depletion in association with other environmental factors. Photochem. Photobiol. Sci..

[109] Purushothaman V.L., Cuomo R.E., Garland C.F., Mackey T.K. (2021). Could age increase the strength of inverse association between ultraviolet B exposure and colorectal cancer?. BMC Public Health.

[110] Berman-Rosa M., Logan J., Ghazawi F.M., Le M., Conte S., Netchiporouk E., Mukovozov I.M., Cyr J., Mourad A., Miller W.H. (2022). Analysis of Geographic and Environmental Factors and Their Association with Cutaneous Melanoma Incidence in Canada. Dermatology.

[111] Najafi E., Khanjani N., Ghotbi M.R., Nejad M.E.M. (2019). The association of gastrointestinal cancers (esophagus, stomach, and colon) with solar ultraviolet radiation in Iran—An ecological study. Environ. Monit. Assess..

[112] Haidari F., Abiri B., Iravani M., Razavi S.M., Vafa M. (2019). The effects of uvb and vitamin D on decreasing risk of colorectal cancer incidence and mortality: A review of the epidemiology, clinical trials, and mechanisms. Nutr. Cancer.

[113] Kiani B., Amin F.H., Bagheri N., Bergquist R., Mohammadi A.A., Yousefi M., Faraji H., Roshandel G., Beirami S., Rahimzadeh H. (2021). Association between heavy metals and colon cancer: An ecological study based on geographical information systems in North-Eastern Iran. BMC Cancer.

[114] Yu P., Xu R., Li S., Coelho M.S., Saldiva P.H., Sim M.R., Abramson M.J., Guo Y. (2022). Associations between long-term exposure to PM2.5 and site-specific cancer mortality: A nationwide study in Brazil between 2010 and 2018. Environ. Pollut..

[115] Sapunar-Zenteno J., Ferrer-Rosende P., Caglevic C. (2021). Incidence of lung cancer and air pollution in boroughs of Chile: An ecological study. ecancermedicalscience.

[116] Gharibvand L., Beeson W.L., Shavlik D., Knutsen R., Ghamsary M., Soret S., Knutsen S.F. (2017). The association between ambient fine particulate matter and incident adenocarcinoma subtype of lung cancer. Environ. Health.

[117] Gariazzo C., Binazzi A., Alfò M., Massari S., Stafoggia M., Marinaccio A. (2021). Predictors of lung cancer risk: An ecological study using mortality and environmental data by municipalities in Italy. Int. J. Environ. Res. Public Health.

[118] Zenteno J.S., Rosende P.F., Manzur B.C., Vega I.S. (2021). Breast cancer incidence and the air pollution level in the communes of Chile: An ecological study. ecancermedicalscience.

[119] Giannoula E., Melidis C., Frangos S., Papadopoulos N., Koutsouki G., Iakovou I. (2021). Ecological study on thyroid cancer incidence and mortality in association with European Union member states’ air pollution. Int. J. Environ. Res. Public Health.

[120] Kedarisetty S., Jones E., Tint D., Soliman A.M. (2019). Air Pollution and Angioedema. Otolaryngol.–Head Neck Surg..

[121] Patella V., Florio G., Palmieri M., Bousquet J., Tonacci A., Giuliano A., Gangemi S. (2020). Atopic dermatitis severity during exposure to air pollutants and weather changes with an Artificial Neural Network (ANN) analysis. Pediatr. Allergy Immunol..

[122] Hüls A., Abramson M.J., Sugiri D., Fuks K., Krämer U., Krutmann J., Schikowski T. (2019). Nonatopic eczema in elderly women: Effect of air pollution and genes. J. Allergy Clin. Immunol..

[123] Miyazaki D., Fukagawa K., Okamoto S., Fukushima A., Uchio E., Ebihara N., Shoji J., Namba K., Shimizu Y. (2020). Epidemiological aspects of allergic conjunctivitis. Allergol. Int..

[124] Dijkhoff I.M., Drasler B., Karakocak B.B., Petri-Fink A., Valacchi G., Eeman M., Rothen-Rutishauser B. (2020). Impact of airborne particulate matter on skin: A systematic review from epidemiology to in vitro studies. Part. Fibre Toxicol..

[125] Abolhasani R., Araghi F., Tabary M., Aryannejad A., Mashinchi B., Robati R.M. (2021). The impact of air pollution on skin and related disorders: A comprehensive review. Dermatol. Ther..

[126] Araviiskaia E., Berardesca E., Bieber T., Gontijo G., Sanchez Viera M., Marrot L., Chuberre B., Dreno B. (2019). The impact of airborne pollution on skin. J. Eur. Acad. Dermatol. Venereol..

[127] Chen Y., He Y.S., Feng Y.T., Wu Z.D., Wang J., Yin K.J., Huang J.X., Pan H.F. (2022). The effect of air pollution exposure on risk of outpatient visits for Sjogren’s syndrome: A time-series study. Environ. Res..

[128] Zhang T.-P., Wang L.-J., Wang S., Wang P., Zhou X.-H., Yang C.-M., Li X.-M. (2022). Exposure to ambient gaseous pollutant and daily hospitalizations for Sjögren’s syndrome in Hefei: A time-series study. Front. Immunol..

[129] Shin J., Lee J., Lee J., Ha E.H. (2019). Association between exposure to ambient air pollution and rheumatoid arthritis in adults. Int. J. Environ. Res. Public Health.

[130] Ho W.C., Chou L.W., Wang R.Y., Doan T.N., Yu H.L., Chou T.H., Liu K.Y., Wu P.C., Shieh S.H. (2022). Association between exposure to ambient air pollution and the risk of rheumatoid arthritis in Taiwan: A population-based retrospective cohort study. Int. J. Environ. Res. Public Health.

[131] Arleevskaya M., Takha E., Petrov S., Kazarian G., Renaudineau Y., Brooks W., Larionova R., Korovina M., Valeeva A., Shuralev E. (2022). Interplay of environmental, individual and genetic factors in rheumatoid arthritis provocation. Int. J. Mol. Sci..

[132] Xi X., Ye Q., Fan D., Cao X., Wang Q., Wang X., Zhang M., Xu Y., Xiao C. (2022). Polycyclic aromatic hydrocarbons affect rheumatoid arthritis pathogenesis via aryl hydrocarbon receptor. Front. Immunol..

[133] Lu M., Ding S., Wang J., Liu Y., An Z., Li J., Song J. (2020). Acute effect of ambient air pollution on hospital outpatient cases of chronic sinusitis in Xinxiang, China. Ecotoxicol. Environ. Saf..

[134] Leland E.M., Vohra V., Seal S.M., Zhang Z., Ramanathan M. (2022). Environmental air pollution and chronic rhinosinusitis: A systematic review. Laryngoscope Investig. Otolaryngol..

[135] Vehof J., Snieder H., Jansonius N., Hammond C.J. (2021). Prevalence and risk factors of dry eye in 79,866 participants of the population-based Lifelines cohort study in the Netherlands. Ocul. Surf..

[136] Akdoğan M.V., Hızal E., Semiz M., Topal Ö., Akkaş H., Kabataş A., Erbek S.S. (2018). The role of meteorologic factors and air pollution on the frequency of pediatric epistaxis. Ear Nose Throat J..

[137] Markeviciute A., Huang-Lung J., Zemaitiene R., Grzybowski A. (2023). A Review of Ambient Air Pollution as a Risk Factor for Posterior Segment Ocular Diseases. J. Clin. Med..

[138] Millen A.E., Dighe S., Kordas K., Aminigo B.Z., Zafron M.L., Mu L. (2023). Air Pollution and Chronic Eye Disease in Adults: A Scoping Review. Ophthalmic Epidemiol..

[139] Wang W., Zhang W., Ge H., Chen B., Zhao J., Wu J., Ma Q. (2022). Association between air pollution and emergency room visits for eye diseases and effect modification by temperature in Beijing, China. Environ. Sci. Pollut. Res..

[140] Gui S.-Y., Wang F., Qiao J.-C., Wang X.-C., Huang Z.-H., Yang F., Hu C.-Y., Tao F.-B., Tao L.-M., Liu D.-W. (2023). Short-term effect of meteorological factors and extreme weather events on daily outpatient visits for dry eye disease between 2013 and 2020: A time-series study in Urumqi, China. Environ. Sci. Pollut. Res..

[141] Deng S.Z., Jalaludin B.B., Antó J.M., Hess J.J., Huang C.R. (2020). Climate change, air pollution, and allergic respiratory diseases: A call to action for health professionals. Chin. Med. J..

[142] Ebi K.L., Vanos J., Baldwin J.W., Bell J.E., Hondula D.M., Errett N.A., Hayes K., Reid C.E., Saha S., Spector J. (2021). Extreme weather and climate change: Population health and health system implications. Annu. Rev. Public Health.

[143] Clayton S. (2021). Climate change and mental health. Curr. Environ. Health Rep..

[144] Liu J., Potter T., Zahner S. (2020). Policy brief on climate change and mental health/well-being. Nurs. Outlook.

[145] Di Cicco M.E., Ferrante G., Amato D., Capizzi A., De Pieri C., Ferraro V.A., Furno M., Tranchino V., La Grutta S. (2020). Climate change and childhood respiratory health: A call to action for paediatricians. Int. J. Environ. Res. Public Health.

[146] Weilnhammer V., Schmid J., Mittermeier I., Schreiber F., Jiang L., Pastuhovic V., Herr C., Heinze S. (2021). Extreme weather events in europe and their health consequences–A systematic review. Int. J. Hyg. Environ. Health.

[147] Wong M.S., Ho H.C., Tse A. (2020). Geospatial context of social and environmental factors associated with health risk during temperature extremes: Review and discussion. Geospat. Health.

[148] Peters R.L., Mavoa S., Koplin J.J. (2022). An overview of environmental risk factors for food allergy. Int. J. Environ. Res. Public Health.

[149] Wu C.-T., Li G.-H., Huang C.-T., Cheng Y.-C., Chen C.-H., Chien J.-Y., Kuo P.-H., Kuo L.-C., Lai F. (2021). Acute exacerbation of a chronic obstructive pulmonary disease prediction system using wearable device data, machine learning, and deep learning: Development and cohort study. JMIR Mhealth Uhealth.

[150] Bashir R.S.E., Hassan O.A. (2019). A One Health perspective to identify environmental factors that affect Rift Valley fever transmission in Gezira state, Central Sudan. Trop. Med. Health.

[151] Li Y.-C., Hsu H.-H.L., Chun Y., Chiu P.-H., Arditi Z., Claudio L., Pandey G., Bunyavanich S. (2021). Machine learning–driven identification of early-life air toxic combinations associated with childhood asthma outcomes. J. Clin. Investig..

[152] Razavi-Termeh S.V., Sadeghi-Niaraki A., Choi S.M. (2021). Asthma-prone areas modeling using a machine learning model. Sci. Rep..

[153] Aimone A.M., Perumal N., Cole D.C. (2013). A systematic review of the application and utility of geographical information systems for exploring disease-disease relationships in paediatric global health research: The case of anaemia and malaria. Int. J. Health Geogr..

[154] Rodriguez-Villamizar L.A., Belalcázar-Ceron L.C., Fernández-Niño J.A., Marín-Pineda D.M., Rojas-Sánchez O.A., Acuña-Merchán L.A., Ramírez-García N., Mangones-Matos S.C., Vargas-González J.M., Herrera-Torres J. (2021). Air pollution, sociodemographic and health conditions effects on COVID-19 mortality in Colombia: An ecological study. Sci. Total Environ..

[155] Bergamaschi R., Ponzano M., Schiavetti I., Carmisciano L., Cordioli C., Filippi M., Radaelli M., Immovilli P., Capobianco M., De Rossi N. (2022). The effect of air pollution on COVID-19 severity in a sample of patients with multiple sclerosis. Eur. J. Neurol..

[156] Schneider R., Masselot P., Vicedo-Cabrera A.M., Sera F., Blangiardo M., Forlani C., Douros J., Jorba O., Adani M., Kouznetsov R. (2022). Differential impact of government lockdown policies on reducing air pollution levels and related mortality in Europe. Sci. Rep..

[157] Beloconi A., Vounatsou P. (2020). Bayesian geostatistical modelling of high-resolution NO_2_ exposure in Europe combining data from monitors, satellites and chemical transport models. Environ. Int..

[158] De Hoogh K., Saucy A., Shtein A., Schwartz J., West E.A., Strassmann A., Puhan M., Roosli M., Stafoggia M., Kloog I. (2019). Predicting fine-scale daily NO_2_ for 2005–2016 incorporating OMI satellite data across Switzerland. Environ. Sci. Technol..

[159] Jacobson L.D.S.V., Oliveira B.F.A.D., Schneider R., Gasparrini A., Hacon S.D.S. (2021). Mortality risk from respiratory diseases due to non-optimal temperature among brazilian elderlies. Int. J. Environ. Res. Public Health.

[160] Gignac F., Righi V., Toran R., Errandonea L.P., Ortiz R., Mijling B., Naranjo A., Nieuwenhuijsen M., Creus J., Basagaña X. (2022). Short-term NO_2_ exposure and cognitive and mental health: A panel study based on a citizen science project in Barcelona, Spain. Environ. Int..

[161] Schaeffer B.A., Bailey S.W., Conmy R.N., Galvin M., Ignatius A.R., Johnston J.M., Keith D.J., Lunetta R.S., Parmar R., Stumpf R.P. (2018). Mobile device application for monitoring cyanobacteria harmful algal blooms using Sentinel-3 satellite Ocean and Land Colour Instruments. Environ. Model. Softw..

[162] Lamy K., Ranaivombola M., Bencherif H., Portafaix T., Toihir M.A., Lakkala K., Arola A., Kujanpää J., Pitkänen M.R.A., Cadet J.-M. (2021). Monitoring Solar Radiation UV Exposure in the Comoros. Int. J. Environ. Res. Public Health.

[163] Mahoney G., Stewart A.G., Kennedy N., Whitely B., Turner L., Wilkinson E. (2015). Achieving attainable outcomes from good science in an untidy world: Case studies in land and air pollution. Environ. Geochem. Health.

[164] Yu L., Liu W., Wang X., Ye Z., Tan Q., Qiu W., Nie X., Li M., Wang B., Chen W. (2022). A review of practical statistical methods used in epidemiological studies to estimate the health effects of multi-pollutant mixture. Environ. Pollut..

[165] Yousaf H.M., Bakhsh K., Masood A. (2023). Nexus of pesticide exposure, personal preventive measures and farm workers’ health safety in cotton production. Hum. Ecol. Risk Assess. Int. J..

[166] Filippini T., Rothman K.J., Cocchio S., Narne E., Mantoan D., Saia M., Goffi A., Ferrari F., Maffeis G., Orsini N. (2021). Associations between mortality from COVID-19 in two Italian regions and outdoor air pollution as assessed through tropospheric nitrogen dioxide. Sci. Total Environ..

[167] Copernicus H. Health. https://www.copernicus.eu/en/use-cases?f%5B0%5D=domain_taxonomy_term_name%3AHealth.

[168] (2022). Copernicus Emergency Management Service—Mapping. https://emergency.copernicus.eu/mapping/.

[169] (2019). CAMS-Downstream—Development of a LQ-WARN-App. https://www.copernicus-user-uptake.eu/user-uptake/details/cams-downstream-development-of-a-lq-warn-app-17.

[170] Yu T., Wang W., Ciren P., Zhu Y. (2016). Assessment of human health impact from exposure to multiple air pollutants in China based on satellite observations. Int. J. Appl. Earth Obs. Geoinf..

[171] Atek S., Pesaresi C., Eugeni M., De Vito C., Cardinale V., Mecella M., Rescio A., Petronzio L., Vincenzi A., Pistillo P. (2022). A Geospatial Artificial Intelligence and satellite-based earth observation cognitive system in response to COVID-19. Acta Astronaut..

[172] (2023). PASYFO: Forecasts of Personal Allergy Symptoms Copernicus. https://www.copernicus.eu/en/use-cases/pasyfo-forecasts-personal-allergy-symptoms.

[173] Earth Observations for Health (EO4HEALTH). http://www.geohealthcop.org/eo4health.

[174] European Health Service Homepage. Service. https://climate.copernicus.eu/european-health-service.

[175] (2020). Copernicus Accelerator. https://accelerator.copernicus.eu/.

[176] Copernicus (2018). airTEXT: Air Quality Information at-a-Glance. https://www.copernicus.eu/en/use-cases/airtext-air-quality-information-glance.

[177] Clark J.M., Schaeffer B.A., Darling J.A., Urquhart E.A., Johnston J.M., Ignatius A.R., Myer M.H., Loftin K.A., Werdell P.J., Stumpf R.P. (2017). Satellite monitoring of cyanobacterial harmful algal bloom frequency in recreational waters and drinking water sources. Ecol. Indic..

[178] Verstraeten W.W., Kouznetsov R., Hoebeke L., Bruffaerts N., Sofiev M., Delcloo A.W. (2021). Modelling grass pollen levels in Belgium. Sci. Total Environ..

