# Supplementary material for: The Effects of Environmental Factors on General Human Health: A Scoping Review
Source: Healthcare (Basel). 2024 Oct 24;12(21):2123. doi: 10.3390/healthcare12212123 (PMC11545045; doi:10.3390/healthcare12212123)
Supplement: Supplementary file 1 [file healthcare-12-02123-s001.zip › supplementary_file_1.pdf]

We also reviewed health-related projects that utilize Copernicus's environmental data. However, these projects are specified for monitoring environmental factors and are not focused on evaluating correlations between environmental factors and health issues.

Copernicus

Since we established that we need continuous monitoring of environmental exposures to improve general health, we suggest and review Copernicus as a solution to provide data services. Copernicus is an Earth observation initiative run by the European Union that explores our planet and its surroundings. It offers information services based on satellite Earth observation and data from the ground (non-space). Copernicus provides unprecedented volumes of past, present, and future environmental data in the form of climate forecasts. This data also encompasses numerical models to provide robust and regularly updated information to customers in the health sector.

Next, we review the most important Copernicus health-related projects available on the Copernicus website [167]. These projects utilize various Copernicus services; e.g., Copernicus emergency management services (CEMS) were used for supporting the management of the COVID-19 emergency in Europe. In one of these projects, satellite images were utilized for monitoring traffic and gatherings, which supported the decision-making process related to the lockdown [168]. Similarly, Copernicus atmosphere management services (CAMS) were used for tracking air pollution changes during COVID-19 restrictions to better understand the effects of weather and air pollution in the pre and post COVID era [156]. LQ-WARN-app is another app that uses CAMS forecasts to warn and inform citizens and public authorities about air pollution concentrations in Germany [169].

In addition, we must acknowledge that when assessing the impact of environmental pollution on human health, using data collected from satellites generally results in lower estimates of the health impact compared to data collected directly on-site (in-situ data). However, remote sensing observations (data collected from satellites) have advantages over in-situ data because they provide better spatial coverage. In other words, satellite data can provide a more comprehensive view of the spatial distribution of the health impact caused by environmental pollution. This broader coverage allows for a better understanding of how environmental pollution affects human health across larger geographic areas [170].

Another project, named Earth Cognitive System for COVID-19 (ECO4CO), made use of space data from satellites (image acquisition, GNSS/EGNOS navigation data), non-space data (the Internet (social media, news, etc.), traffic data (mobile, automotive), and medical data (equipment stock, hospitalizations) to support decision-making by Italian civil protection and health authorities; in addition, this data provided rapid insights and evidence of geo-localized events that demonstrated the potential to impact the evolution of outbreaks [171].

A similar app was developed by the Finnish Meteorological Institute, the Medical University of Vienna, and the University of Latvia; this app, which is an operational pollen product referred to as the PASYFO application. By using the Copernicus vegetation index and land surface temperature, birch pollen outbreaks are monitored and forecasted by SEN4POL (Towards a Sentinel-based pollen information service) for protecting asthma-allergy patients. Forecasting allergy risk for individuals with pollen allergies is important information that can aid in disease self-management and symptom severity reduction [172].

Likewise, with the goal of using Earth observational data to enhance health decision-making at the international, regional, national, and district levels, the Earth Observations for Health (EO4HEALTH) initiative will act as a global network of governments, organizations, and observers. The overall objective of the EO4HEALTH initiative is to support the systematic gathering, analysis, and application of relevant information about risky areas that assist in the development of strategic responses to foresee risks, opportunities, and their evolution and communicate options for the purposes of decision-making and response [173].

Another project anticipated that vector habitats and the geographic ranges of vector-borne pathogens such as malaria, dengue fever, and yellow fever, would expand as a result of climate change. Utilizing the potential of the Copernicus climate emergency monitoring (C3S) infrastructure, a climate-driven vector-borne disease risk assessment platform enables planning efficient management strategies and emphasizes taking advantage of how dependent vectors, like mosquitoes, are on the environment for their survival [174].

Similarly, one of the use cases of Copernicus services is UV-Bodyguard Smart in the sun. Powered by CAMS-UV-Product, it deals with the problem of being exposed to too much sun or not enough sun exposure. Sun exposure is vital for vitamin D but too much exposure can cause sunburn, skin cancer, and other health issues. This app forecasts and tracks UV exposure and even recommends the best UV protection [175]. Another useful app is Riga airTEXT, which produces air quality forecasts using CAMS so the general population can be ready for brief periods of poor air quality in advance. People who have asthma or heart disease can take proactive measures, such as keeping an inhaler handy or staying away from physically demanding outdoor activities [176].

The literature survey in this review also included articles that utilized satellite data using the Copernicus website. Rodriguez et al. determined the association between $PM_{2.5}$ exposures and COVID-19 mortality [98]. The authors retrieved $PM_{2.5}$ concentrations from CAMS Reanalysis (CAMSRA) and CAMS Near Real-Time (CAMSNRT) because of the unavailability of air pollution data for medium and smaller cities of the country. In [155], the CAMS air quality model findings were used by Roberto et al. to calculate $PM_{2.5}$ ground level values in accordance with the location of COVID-19 exposure as stated by the patients so that exposure measurement is specific to an individual's location. Schneider et al. [156] used an ensemble of six state-of-the-art chemistry-transport numerical forecast models that are a part of CAMS's continuous air quality monitoring service to simulate air pollution concentrations under two emission scenarios: one with COVID-19 restrictions and the other without COVID-19 restrictions. They also applied a spatiotemporal Bayesian non-linear mixed effect model to quantify the changes in pollutant concentrations associated with the stringency indices of individual policy measures. They aimed to observe the effects of the lockdown on air pollution levels and CAMS provided the solution.

Beloconi et al. [157] also used CAMS, a reanalysis dataset of the atmospheric composition produced by the European Centre for Medium-range Weather Forecasts (ECMWF). They also used CAMS-Ensemble simulations to measure annual averages of NO_2 concentration in $\mu g/m^3$ and used a Bayesian geostatistical model for the estimation of air pollution exposure at high spatial resolution. De et al. [158] demonstrated that by using earth observation data, we can develop robust models for forecasting and model temporal (daily) variations of several pollutants, such as NO_2. These predictions can help with

medical research. Similarly, Jacobson et al. [159] used Copernicus ERA5-Land reanalysis to extract daily mean temperature and relative humidity for their study to find an association between daily average temperature and respiratory mortality among elderly Brazilians.

Copernicus Sentinel-3 satellite ocean and land colour instruments were used by the cyanobacteria assessment network (CyAN) mobile device application (app) in research published by Schaeffer et al. [161]. This application performed real-time monitoring of water quality, made assessments and transmitted alerts if there was a potential problem related to harmful algal blooms. Similar to this case, Clark et al. suggested that Copernicus program Sentinel 1 and 2 sensors have a higher spatial resolution [177]. A Belgian study by Verstraeten et al. utilized [178] European MACC-III grassland maps and CLMS (2015) land-use maps and produced an updated map of the grasslands of Belgium. They then applied a chemistry transport model for a better forecast with updated seasonal maps. While trying to find associations between NO_2 concentration levels and COVID-19 mortality in Italy, Filippini et al. [166] used Sentinel-5P satellites of the European space agency Copernicus Earth Observation Program. In addition, for temperature data, they used the ERAS model reanalysis of the European Centre for Medium-Range Weather Forecasts website (ECMWF).

Similarly, ECMWF was used for monitoring solar radiation UV exposure in a study from Comoros by Lamy et al. [162]. While using CAMS and a tropospheric UV model, they were able to find various measurements and estimates that allowed them to quantify, evaluate, and monitor the health risk associated with UV radiation exposure in that region and to help understand how cloud cover influences the variability of UV radiation on the ground. In [175], while measuring mean NO_2 concentration exposure for participants who shared their location data, Gignac et al. used air quality estimates from the CAMS when the participant would move outside Barcelona or away from ground NO_2 exposure measurement stations.

References

[98] Y. Ma, J. Zhou, S. Yang, Y. Zhao, and X. Zheng, "Assessment for the impact of dust events on measles incidence in western China," Atmospheric Environment 2017, 157, 1–9., 2017.

[155] N. ; et al. T. effect of air pollution on C.-19 severity in a sample of patients with multiple sclerosis 2022. Roberto B. ;. Marta, P. ;. Irene, S. ;. Luca, C. ;. Cinzia, C. ;. Massimo, F. ;. Marta, R. ;. Paolo, I. ;. Marco, C. ;. De Rossi, "Title Missing," Journal Missing.

[156] R. Schneider et al., "Differential impact of government lockdown policies on reducing air pollution levels and related mortality in Europe," Scientific reports 2022, 12, 1–13., 2022.

[157] A. Beloconi and P. Vounatsou, "Bayesian geostatistical modelling of high-resolution NO2 exposure in Europe combining data from monitors, satellites and chemical transport models," Environment international 2020, 138, 105578., 2020.

[158] K. De Hoogh et al., "Predicting fine-scale daily NO2 for 2005–2016 incorporating OMI satellite data across Switzerland," Environmental science & technology 2019, 53, 10279–10287., 2019.

[159] L. d. S. V. Jacobson, B. F. A. d. Oliveira, R. Schneider, A. Gasparrini, and S. d. S. Hacon, "Mortality risk from respiratory diseases due to non-optimal temperature among brazilian elderlies," International journal of environmental research and public health 2021, 18, 5550., 2021.

[161] B. A. Schaeffer et al., "Mobile device application for monitoring cyanobacteria harmful algal blooms using Sentinel-3 satellite Ocean and Land Colour Instruments," Environmental modelling & software 2018, 109, 93 103., 2018.

[162] K. Lamy et al., "Monitoring Solar Radiation UV Exposure in the Comoros," International journal of environmental research and public health 2021, 18, 10475., 2021.

[166] T. Filippini et al., "Associations between mortality from COVID-19 in two Italian regions and outdoor air pollution as assessed through tropospheric nitrogen dioxide," Science of the Total Environment 2021, 760, 143355., 2021.

[167] H. Copernicus, "Available at: https://www.copernicus.eu/en/use-cases?f%5B0%5D=domain_taxonomy_term_name%3AHealth (Accessed: February 16, 2023)," .

[168] C. E. M. S. (2022) C. E.- Mapping, "Available at: https://emergency.copernicus.eu/mapping/ (Accessed: February 16, 2023)," ..

[169] C. – development of a L. (2019) F. P. A. on C. U. Uptake (FPCUP), "Available at: user-uptake.eu/user-uptake/details/cams-downstream development-of-a-lq-warn-app-17 (Accessed: February 16, 2023)," ..

[170] T. Yu, W. Wang, P. Ciren, and Y. Zhu, "Assessment of human health impact from exposure to multiple air pollutants in China based on satellite observations," International journal of applied earth observation and geoinformation 2016, 52, 542–553., 2016.

[171] S. Atek et al., "A Geospatial Artificial Intelligence and satellite-based earth observation cognitive system in response to COVID-19," Acta Astronautica 2022., 2022.

[172] (2023). PASYFO: Forecasts of personal allergy symptoms Copernicus. Available at: (Accessed: February 16, "Title Missing," Journal Missing.

[173] E. G. H. CoP, "Available at: http://www.geohealthcop.org/eo4health (Accessed: February 16, 2023),"

[174] E. H. S. Homepage, "Available at: https://climate.copernicus.eu/european-health-service (Accessed: February 16, 2023)," ..

[175] U. by A. (2020) C. Accelerator, "Available at: https://accelerator.copernicus.eu/(Accessed: February 16, 2023)," ..

[176] A. A. Q. I. at-a-glance (27 F. 2018) Copernicus, "Available at: https://www.copernicus.eu/en/use-cases/airtext-air-quality-information-glance (Accessed: February 16, 2023)," ..

[177] J. M. Clark et al., "Satellite monitoring of cyanobacterial harmful algal bloom frequency in recreational waters and drinking water sources," Ecological indicators 2017, 80, 84–95., 2017.

[178] W. W. Verstraeten, R. Kouznetsov, L. Hoebeke, N. Bruffaerts, M. Sofiev, and A. W. Delcloo, "Modelling grass pollen levels in Belgium," Science of The Total Environment 2021, 753, 141903., 2021.
