# Supplementary material for: The Effects of Environmental Factors on General Human Health: A Scoping Review
Source: Healthcare (Basel). 2024 Oct 24;12(21):2123. doi: 10.3390/healthcare12212123 (PMC11545045; doi:10.3390/healthcare12212123)
Supplement: Supplementary file 1 [file healthcare-12-02123-s001.zip › Table S1.pdf]

Title: The Effects of Environmental Factors on General Human Health: Scoping Review

Ref	Year of publication	Article type	Target Disease									Copernicus / Satellite data	Target Environmental Factor										
			Respiratory	Cardiovascular	Neurological and Psychological	Skin diseases	Other/Miscellaneous	Renal and urogenital	Metabolic and gastrointestinal	Oncological	Infectious diseases		Air Quality/Pollution	Greenness	Humidity	Temperature	Precipitation	UV radiation	Climate Change	Interplay	Wind speed	Atmospheric pressure	Ozone
[126]	2013	Review									✓	✓											
[91]	2013	Letter to the Editor									✓				✓	✓							
[13]	2013	Research article	✓							✓	✓		✓										
[93]	2014	Research article									✓				✓	✓					✓	✓	
[94]	2014	Research article									✓				✓	✓							
[10]	2015	Research article								✓						✓		✓					
[166]	2015	Research article								✓						✓		✓					✓
[59]	2015	Research article		✓												✓							
[56]	2015	Research article		✓									✓										
[34]	2015	Research article	✓										✓			✓							
[131]	2016	Research article										✓	✓										
[74]	2016	Research article						✓								✓							
[50]	2017	Review		✓									✓										
[75]	2017	Research article			✓								✓	✓									
[17]	2017	Research article							✓				✓										
[110]	2017	Research article								✓			✓										
[92]	2017	Research article									✓				✓	✓					✓	✓	
[89]	2017	Research article									✓					✓					✓		
[90]	2017	Research article									✓					✓	✓						
[177]	2017	Research article									✓	✓											
[16]	2018	Review		✓									✓	✓	✓	✓				✓			
[45]	2018	Research article	✓													✓							
[61]	2018	Review		✓											✓	✓				✓			
[48]	2018	Research article		✓									✓										
[2]	2018	Review		✓									✓										
[164]	2018	Research article					✓						✓		✓	✓						✓	
[32]	2018	Review	✓										✓										
[68]	2018	Review	✓						✓														
[160]	2018	Research article										✓											
[57]	2018	Review		✓									✓										
[141]	2018	Research article									✓	✓											
[137]	2018	Copernicus Project										✓	✓										
[41]	2019	Research article	✓	✓									✓	✓						✓			
[51]	2019	Review		✓									✓										
[80]	2019	Research article			✓											✓							
[101]	2019	Research article			✓								✓										
[72]	2019	Research article							✓					✓									
[106]	2019	Research article							✓	✓								✓					
[107]	2019	Review								✓								✓					
[153]	2019	Research article				✓							✓										
[123]	2019	Research article									✓	✓		✓									
[71]	2019	Research article	✓	✓	✓				✓				✓										
[30]	2019	Research article	✓										✓										
[67]	2019	Review							✓				✓					✓					
[22]	2019	Research article	✓	✓									✓							✓			
[38]	2019	Research article	✓										✓										
[171]	2019	Review				✓							✓										
[176]	2019	Research article					✓						✓										
[139]	2019	Research article										✓	✓										
[130]	2019	Copernicus Project										✓	✓										
[63]	2020	Review							✓		✓		✓										
[43]	2020	Review	✓	✓					✓		✓					✓			✓				
[53]	2020	Research article		✓									✓										
[54]	2020	Review		✓									✓										
[60]	2020	Review		✓									✓	✓									
[77]	2020	Research article			✓								✓										
[78]	2020	Research article			✓								✓										
[167]	2020	Review				✓							✓						✓				✓

Title: The Effects of Environmental Factors on General Human Health: Scoping Review

Ref	Year of publication	Article type	Target Disease									Copernicus / Satellite data	Target Environmental Factor										
[168]	2020	Review				✓							✓					✓	✓				✓
[79]	2020	Research article			✓								✓										
[82]	2020	Research article			✓								✓		✓	✓					✓		
[83]	2020	Research article			✓										✓	✓							
[85]	2020	Review			✓														✓				
[99]	2020	Research article									✓		✓										
[102]	2020	Research article			✓								✓		✓	✓	✓						
[113]	2020	Research article								✓			✓										
[117]	2020	Research article			✓														✓				
[118]	2020	Review	✓												✓	✓	✓		✓	✓			
[70]	2020	Review							✓				✓										
[44]	2020	Research article	✓										✓		✓	✓	✓						
[55]	2020	Research article		✓									✓										
[64]	2020	Research article							✓				✓										
[26]	2020	Research article	✓										✓							✓			
[66]	2020	Research article							✓				✓			✓							
[84]	2020	Review			✓														✓				
[114]	2020	Review	✓										✓						✓				
[149]	2020	Research article						✓					✓	✓									
[120]	2020	Review														✓				✓			
[147]	2020	Research article						✓					✓										
[138]	2020	Research article										✓	✓										
[144]	2020	Research article					✓						✓										
[136]	2020	Copernicus Project										✓						✓					
[134]	2020	Copernicus Project									✓	✓	✓										
[161]	2021	Research article					✓						✓										
[12]	2021	Review	✓										✓										
[31]	2021	Research article	✓										✓										
[65]	2021	Research article							✓				✓										
[28]	2021	Research article	✓								✓		✓							✓			
[170]	2021	Review				✓							✓										
[36]	2021	Research article	✓	✓									✓										
[49]	2021	Research article	✓	✓									✓	✓	✓	✓							
[148]	2021	Research article						✓					✓										
[47]	2021	Review		✓									✓										
[58]	2021	Research article		✓							✓				✓	✓							
[40]	2021	Research article	✓										✓	✓									
[18]	2021	Research article			✓								✓										
[86]	2021	Research article			✓														✓				
[39]	2021	Review	✓	✓	✓								✓	✓	✓		✓			✓	✓		
[104]	2021	Research article								✓								✓					
[108]	2021	Research article								✓			✓										
[109]	2021	Research article								✓			✓										
[111]	2021	Research article								✓			✓										
[112]	2021	Research article								✓			✓										
[115]	2021	Review																	✓				
[156]	2021	Review						✓					✓										
[116]	2021	Review			✓														✓				
[119]	2021	Review	✓	✓	✓								✓						✓	✓			
[122]	2021	Research article	✓										✓		✓	✓							
[52]	2021	Research article		✓									✓										
[100]	2021	Research article									✓		✓										
[33]	2021	Research article	✓										✓	✓									
[124]	2021	Research article	✓										✓										
[35]	2021	Research article	✓										✓										
[125]	2021	Research article	✓										✓		✓	✓	✓						
[73]	2021	Research article							✓					✓									
[76]	2021	Review			✓									✓									
[62]	2021	Research article	✓	✓											✓	✓							
[95]	2021	Research article									✓					✓							
[96]	2021	Research article									✓	✓	✓										

Title: The Effects of Environmental Factors on General Human Health: Scoping Review

Ref	Year of publication	Article type	Target Disease									Copernicus / Satellite data	Target Environmental Factor										
[98]	2021	Research article									✓	✓	✓										
[140]	2021	Research article	✓									✓				✓							
[143]	2021	Copernicus Project										✓	✓										
[42]	2021	Research article				✓							✓										
[150]	2021	Review						✓					✓										
[158]	2021	Copernicus Project										✓						✓					
[14]	2022	Research article	✓	✓							✓		✓										
[21]	2022	Research article	✓								✓		✓										
[23]	2022	Research article	✓										✓			✓							
[151]	2022	Review			✓			✓					✓										
[46]	2022	Review		✓									✓										
[25]	2022	Research article	✓										✓		✓	✓				✓			
[27]	2022	Research article	✓										✓		✓							✓	
[15]	2022	Research article	✓											✓						✓			✓
[37]	2022	Research article	✓										✓			✓							
[19]	2022	Research article			✓								✓										
[169]	2022	Research article						✓					✓										
[81]	2022	Research article			✓									✓									
[87]	2022	Review			✓									✓					✓				
[88]	2022	Review									✓								✓				
[24]	2022	Research article	✓										✓							✓			
[105]	2022	Research article								✓				✓		✓	✓	✓					
[9]	2022	Research article									✓		✓										
[29]	2022	Review	✓		✓								✓	✓									
[69]	2022	Research article							✓							✓			✓				
[121]	2022	Review																		✓			
[103]	2022	Research article									✓				✓	✓							
[11]	2022	Research article									✓		✓		✓	✓							
[8]	2022	Research article	✓	✓									✓						✓				
[97]	2022	Research article									✓	✓	✓										
[129]	2022	Copernicus Project									✓	✓	✓										
[132]	2022	Research article									✓	✓											
[145]	2022	Research article			✓								✓										
[174]	2022	Research article					✓						✓										
[175]	2022	Research article					✓						✓										
[154]	2022	Research article					✓						✓										
[155]	2022	Review					✓						✓							✓			
[157]	2022	Research article					✓						✓										
[159]	2022	Review					✓						✓										
[173]	2022	Research article						✓					✓										
[162]	2022	Research article					✓						✓			✓				✓			
[178]	2023	Review					✓						✓										
[172]	2023	Research article						✓					✓										
[142]	2023	Review					✓						✓										
[141]	2023	Research article					✓								✓	✓						✓	
[133]		Copernicus Project	✓									✓	✓										
[135]		Copernicus Project									✓	✓							✓				

Table S1: Summary of the reviewed manuscripts focused on the effect of major environmental factors on general human health: The diseases discussed under the influence of environment, manuscripts containing satellite or Copernicus data, the environmental factors examined in the context of human health.
